# Supplementary material for: Systems modeling and uncertainty quantification of AMP-activated protein kinase signaling
Source: NPJ Syst Biol Appl. 2025 Oct 14;11:113. doi: 10.1038/s41540-025-00588-w (PMC12521545; doi:10.1038/s41540-025-00588-w)
Supplement: Supplementary file 1 — Supplementary Information [file 41540_2025_588_MOESM1_ESM.pdf]

Supplementary Information
Systems modeling and uncertainty quantification of AMP-activated protein kinase signaling

Nathaniel Linden-Santangeli,[1] Jin Zhang,[2] Boris Kramer,[1*] Padmini Rangamani[2,1*]

[1]Department of Mechanical and Aerospace Engineering, University of California San Diego,
[2]Department of Pharmacology, University of California San Diego,

*To whom correspondence should be addressed; E-mail: bmkramer@ucsd.edu; prangamani@ucsd.edu.

Supplementary Notes

Supplementary Note 1: Two-reaction models

To further investigate how the choice of kinetic formulation affects parameter identifiability, we examined models of a two-reaction loop that was inspired by a phosphorylation-dephosphorylation loop. The models represented the reactions

$$\mathrm{S} + \mathrm{E}_1 \underset{k_{f,1}}{\overset{k_{r,1}}{\rightleftharpoons}} \mathrm{E}_1\mathrm{S} \xrightarrow{k_{\mathrm{cat},1}} \mathrm{P} + \mathrm{E}_1$$

$$\mathrm{P} + \mathrm{E}_2 \underset{k_{f,2}}{\overset{k_{r,2}}{\rightleftharpoons}} \mathrm{E}_2\mathrm{S} \xrightarrow{k_{\mathrm{cat},2}} \mathrm{pS} + \mathrm{E}_2,$$

where S is the substrate, E_1 and E_2 are the enzymes, and P is the product. The product of the first reaction is the substrate of the second P and the product of the second reaction is the original substrate S. To investigate the effects of the kinetic formulation, we constructed nine sets of models that represented the two-reaction systems. The models differed in the kinetic formulation used to represent either reaction, specifically mass action, Michaelis-Menten, or Hill-type. For example, one of the models employed mass action kinetics for both reactions, while another used mass action for the first reaction and the Michaelis-Menten equation for the second. Supplementary Table S2 summarizes the models and the identifiability results.

Supplementary Note 2: AMPK model details

In this section, we provide additional rationale and derivations for the six AMPK signaling models. Unless otherwise noted, all relevant assumptions are outlined in the manuscript.

2.1 Module 1: Cellular metabolism and energy stress

We adapted the simplified metabolism models from Coccimiglio et al. [1] and Lueng et al. [2]. Our metabolism model includes phenomenological representations of oxidative phosphorylation, glycolysis, ATP hydrolysis, adenylate kinase, and creatine kinase. First, we represented ATP generation by oxidative phosphorylation with a first-order irreversible reaction from ADP to ATP, which we modeled with the Hill-type equation originally derived in [3]. Second, we represented glycolysis with an irreversible first-order reaction from ADP to ATP, which we modeled with mass action kinetics. Third, we represented net ATP consumption due to hydrolysis with a single hydrolysis reaction from ATP to

ADP, which we modeled with mass action kinetics. Fourth, we included both the adenylate and the creatine kinase reactions to ensure the correct buffering of adenine ratios [4]. The adenylate kinase reaction is

$$\mathrm{ATP} + \mathrm{AMP} \underset{J_{\mathrm{AK,rev}}}{\overset{J_{\mathrm{AK,for}}}{\rightleftharpoons}} 2\mathrm{ADP},$$

which we modeled with a bi-bi reaction mechanism originally derived in [4]. Finally, the reversible creatine kinase reaction is

$$\mathrm{ADP} + \mathrm{PCr} \underset{J_{\mathrm{CK,rev}}}{\overset{J_{\mathrm{CK,for}}}{\rightleftharpoons}} \mathrm{ATP} + \mathrm{Cr},$$

which we modeled with a Cleland bi-bi reaction that was originally derived in [4]. Supplementary Table S3 lists the fluxes, J_{OhPhos}, J_{Gly}, etc., for these reactions.

The associated differential equations for ATP, ADP, AMP, and PCr, are defined as,

$$\mathrm{d[AMP]}/\mathrm{d}t = -J_{\mathrm{AK}} \tag{1a}$$

$$\mathrm{d[ADP]}/\mathrm{d}t = -J_{\mathrm{glycolysis}} - J_{\mathrm{OxPhos}} + J_{\mathrm{hydro}} + J_{\mathrm{CK}} + 2J_{\mathrm{AK}} \tag{1b}$$

$$\mathrm{d[ATP]}/\mathrm{d}t = J_{\mathrm{glycolysis}} + J_{\mathrm{OxPhos}} - J_{\mathrm{hydro}} - J_{\mathrm{CK}} - J_{\mathrm{AK}} \tag{1c}$$

$$\mathrm{d[PCr]}/\mathrm{d}t = J_{\mathrm{CK}}. \tag{1d}$$

Throughout this work, we assume that the initial concentrations of AMP, ADP, and ATP are 7.03 mM, 1.11 mM, and 7.9×10^{-2} mM, respectively. Supplementary Figure S1A shows transient AMP, ADP, and ATP concentrations from an arbitrary initial condition to the steady state taken as the initial condition in this work. The cellular metabolism model is the same across all models that we develop. Supplementary Table S4 provides values for all of the Module 1 parameters. All parameter values were taken from sources, except for the rate of ATP production by glycolysis. We derived the glycolysis rate $k_{\mathrm{glycolysis}}$ roughly on experimental measurements of glycolytic flux from Salti et al. [5] Figure 2. In MEF cells, those authors measured a flux of 9.16 pmol/ATP/10^3 cells, which we converted to mM/s by assuming a cellular volume of 4000 $\mu\mathrm{m}^3$, which gives approximately 22 mM/s. We divided by twice initial concentration of ATP (7.03 mM) and rounded down to yield k_{gly} =1.5 1/s.

2.2 Module 2: Adenine nucleotide binding and AMPK phosphorylation

The models include adenine nucleotide binding to AMPK, phosphorylation of AMPK, and dephosphorylation of AMPK. In this section, we describe additional model assumptions and outline the nonessential and essential activation/inhibition mechanisms used in the models.

Adenine nucleotide binding We assumed that adenine nucleotide binding kinetics do not vary depending on the phosphorylation or binding state of AMPK. For example, in the models, the reactions

$$\mathrm{AMP} + \mathrm{AMPK} \overset{K^{\mathrm{AMPK}}_{\mathrm{d,AMP}}}{\rightleftharpoons} \mathrm{AMP–AMPK},$$

$$\mathrm{AMP} + \mathrm{pAMPK} \overset{K^{\mathrm{AMPK}}_{\mathrm{d,AMP}}}{\rightleftharpoons} \mathrm{AMP–pAMPK}, \quad \text{and}$$

$$\mathrm{AMP} + \mathrm{LKB1–AMPK} \overset{K^{\mathrm{AMPK}}_{\mathrm{d,AMP}}}{\rightleftharpoons} \mathrm{AMP–LKB1–AMPK}$$

all occur with the same kinetics. We used the binding coefficients from [6] as initial values when constructing prior densities for these parameters.

AMPK phosphorylation by upstream kinases LKB1 and CaMKK2 We assumed that AMPK phosphorylation by LKB1 and CaMKK2 occurs as a standard enzyme-mediated reaction [7]. For example, phosphorylation by LKB1 would be described as

$$\mathrm{AMPK} + \mathrm{LKB1} \overset{K^{\mathrm{AMPK}}_{\mathrm{d,LKB1}}}{\rightleftharpoons} \mathrm{LKB1–AMPK} \xrightarrow{k^{\mathrm{AMPK}}_{\mathrm{phos,LKB1}}} \mathrm{pAMPK} + \mathrm{LKB1}.$$

We assume that the activity of LKB1 is constant and does not depend on the input stimuli. Whereas we assume that CaMKK2 activity depends on the calcium concentration through interactions with the calcium-binder calmodulin. We describe the calcium-calmodulin-CaMKK2 in detail below.

In order to capture the substrate-mediated effects of AXP binding, we assumed that AMP and ADP act as either essential activators (Models 1 and 2) or nonessential activators (Models 4–6) of upstream kinases. Essential activation assumes that the activator must be bound to the substrate for the reaction to proceed [7]. For example,

$$\text{AMP} + \text{AMPK} \underset{k_{\text{on,AMP}}}{\overset{k_{\text{off,AMP}}}{\rightleftharpoons}} \text{AMP-AMPK}$$

$$\text{AMP-AMPK} + \text{LKB1} \underset{k_{\text{on,LKB1}}}{\overset{k_{\text{off,LKB1}}}{\rightleftharpoons}} \text{LKB1-AMP-AMPK}$$

$$\text{LKB1-AMP-AMPK} \xrightarrow{k_{\text{phos,LKB1}}} \text{LKB1} + \text{AMP-pAMPK},$$

assumes that AMP is the essential activator of LKB1-induced phosphorylation. Alternatively, nonessential activation assumes that the activator increases the reaction rate or decreases the K_d between the enzyme and substrate [7]. For example, in the reaction

$$\text{AMP} + \text{AMPK} \underset{k_{\text{on,AMP}}}{\overset{k_{\text{off,AMP}}}{\rightleftharpoons}} \text{AMP-AMPK} \tag{2}$$

$$\text{AMPK} + \text{LKB1} \underset{k_{\text{on,LKB1}}}{\overset{k_{\text{off,LKB1}}}{\rightleftharpoons}} \text{LKB1-AMPK} \tag{3}$$

$$\text{AMP-AMPK} + \text{LKB1} \underset{k_{\text{on,LKB1}}}{\overset{\alpha_{\text{LKB1}} k_{\text{off,LKB1}}}{\rightleftharpoons}} \text{LKB1-AMP-AMPK} \tag{4}$$

$$\text{LKB1-AMPK} \xrightarrow{k_{\text{phos,LKB1}}} \text{LKB1} + \text{pAMPK} \tag{5}$$

$$\text{LKB1-AMP-AMPK} \xrightarrow{k_{\text{phos,LKB1}}} \text{LKB1} + \text{AMP-pAMPK} \tag{6}$$

the activator AMP decreases the K_d by a factor of $\alpha_{\text{LKB1}} \leq 1$.

We utilize the model of calcium-induced CaMKK2 activity from [8]. Specifically, we assume that CaMKK2 is activated by calcium-bound calmodulin (CaCaM). Calcium binds to calmodulin (CaM) via the reaction

$$3\text{Ca}^{2+} + \text{CaM} \underset{k_{\text{OnCaM}}}{\overset{k_{\text{OffCaM}}}{\rightleftharpoons}} \text{CaCaM}.$$

Next, CaMKK2 is phosphorylated by the CaCaM complex with the reaction

$$\text{CaMKK2} \rightarrow \text{pCaMKK2},$$

with the rate

$$J_{\text{pCaMKK}} = \frac{k_{\text{PhosCaM}}[\text{CaCaM}]^4[\text{CaMKK}]}{K_{\text{m,CaM}}^2 + [\text{CaCaM}]^4}.$$

Active CaMKK2 is dephosphorylated by a first-order reaction, with rate

$$J_{\text{pCaMKK}_{\text{dephos}}} = k_{\text{DephosCaMKK}}[\text{pCaMKK2}].$$

We follow the same strategy as described in the previous paragraph to capture substrate-mediated effects on CaMKK2-induced phosphorylation of AMPK.

AMPK dephosphorylation Similar to phosphorylation, we assumed that dephosphorylation of phosphorylated pAMPK occurs as a standard enzyme-mediated reaction. In Models 1 and 2, we assumed that AMP and ADP act as essential inhibitors of the dephosphorylation reaction, where AMP-pAMPK and ADP-pAMPK are not dephosphorylated. In Models 3–6, we assumed that AMP and ADP acts as nonessential inhibitors of the dephosphorylation reaction, either increasing the K_d (Models 3 and 4) or decreasing the reaction rate (Models 5 and 6).

Supplementary Note 3: Predictive prior elicitation and prior refinement

We encountered two key challenges in constructing prior densities for model parameters: (i) choosing priors that yield reasonable predictions when little prior information is available and (ii) assigning values to nonidentifiable parameters that we fix before estimation. Here, we took an approach that combined predictive prior elicitation with iterative updating of fixed parameters. Predictive prior elicitation is a qualitative approach that allows us to adjust the parameters of the prior densities such that the prior predictive distribution appears reasonable [9]. This allows us to refine our prior beliefs without utilizing any data. Briefly, the prior predictive distribution shows the distribution of data generated by the model given assumed prior densities [10]. In the remainder of this section, we outline the process used to refine prior densities and select values for non-identifiable parameters, using Model 2 as an example.

We began by using direct prior elicitation to choose default priors for uncertain parameters by specifying a range of plausible values (see Methods) [9]. Using these priors for Model 2, we observed that the prior predictive density was unreasonable, because all of the predicted simulations were constantly zero (Supplementary Figure S3A). We hypothesized that the values of the nonidentifiable parameters, which we fixed to nominal values, could account for the observed behavior. To address this, we varied each of the five nonidentifiable parameters independently over several orders of magnitude and simulated the corresponding prior predictive densities. Supplementary Figure S3B shows the cumulative density functions of the final value of the predicted sensor response. We found that only k_{AMPK} affected the predictions, so we updated the nominal value for that parameter to 0.192 because that value yielded more reasonable predictions. We repeated this process for the remaining parameters and found that we only needed one additional iteration of updates, because only k_{CaMKK} affected predictions (Supplementary Figure S3C). Given that we were satisfied with the updated nominal values for nonidentifiable parameters, we next chose to refine the priors using predictive prior elicitation. Here, we simulated the prior density and iteratively updated the hyperparameters of the priors to yield more reasonable predictions. We refer the reader to the tutorial in the Preliz Python package, at the following link `https://preliz.readthedocs.io/en/latest/examples/gallery/predictive_explorer.html` for more details. Supplementary Figure S3D shows the resulting prior predictive distribution and Supplementary Figure S3E shows the densities of the final value in the simulation and the time to half max. We repeated this process for all six AMPK models (Supplementary Figure S2).

Supplementary Figures

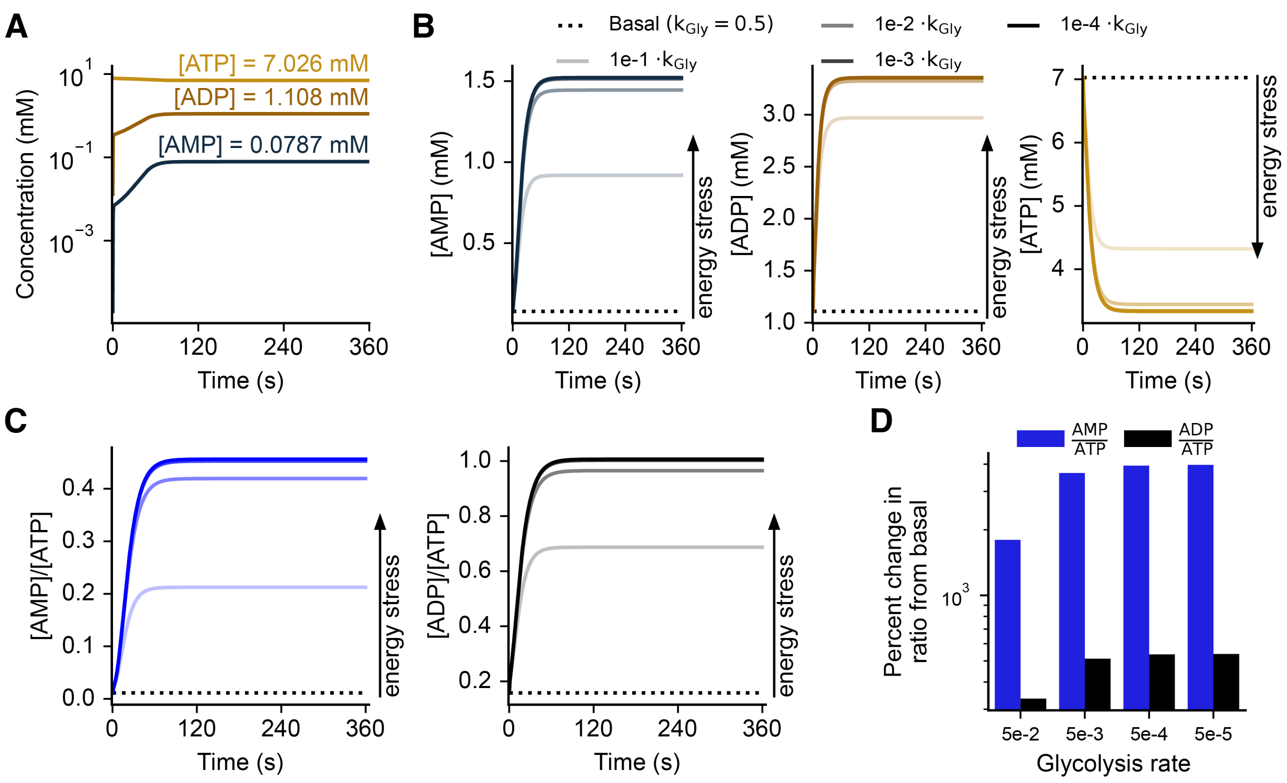

Supplementary Figure S1: **Simulated energy stress with decreased glycolytic ATP production increases the AMP/ATP and ADP/ATP ratios**. (**A**) AMP, ADP, and ATP transients from arbitrary initial conditions to steady state values with basal ATP production. (**B**) Effect of energy stress due to decreasing the rate of glycolytic ATP production on AMP, ADP, and ATP concentrations over time. The rate of glycolysis is changed from the basal value at $t = 0$. (**C**) Effect of energy stress on transient AMP/ATP (blue) and ATP/ADP (black) ratios. Stimuli are the same as in B. (**D**) Percent change in AMP/ATP and ADP/ATP ratios from basal due to increased energy stress.

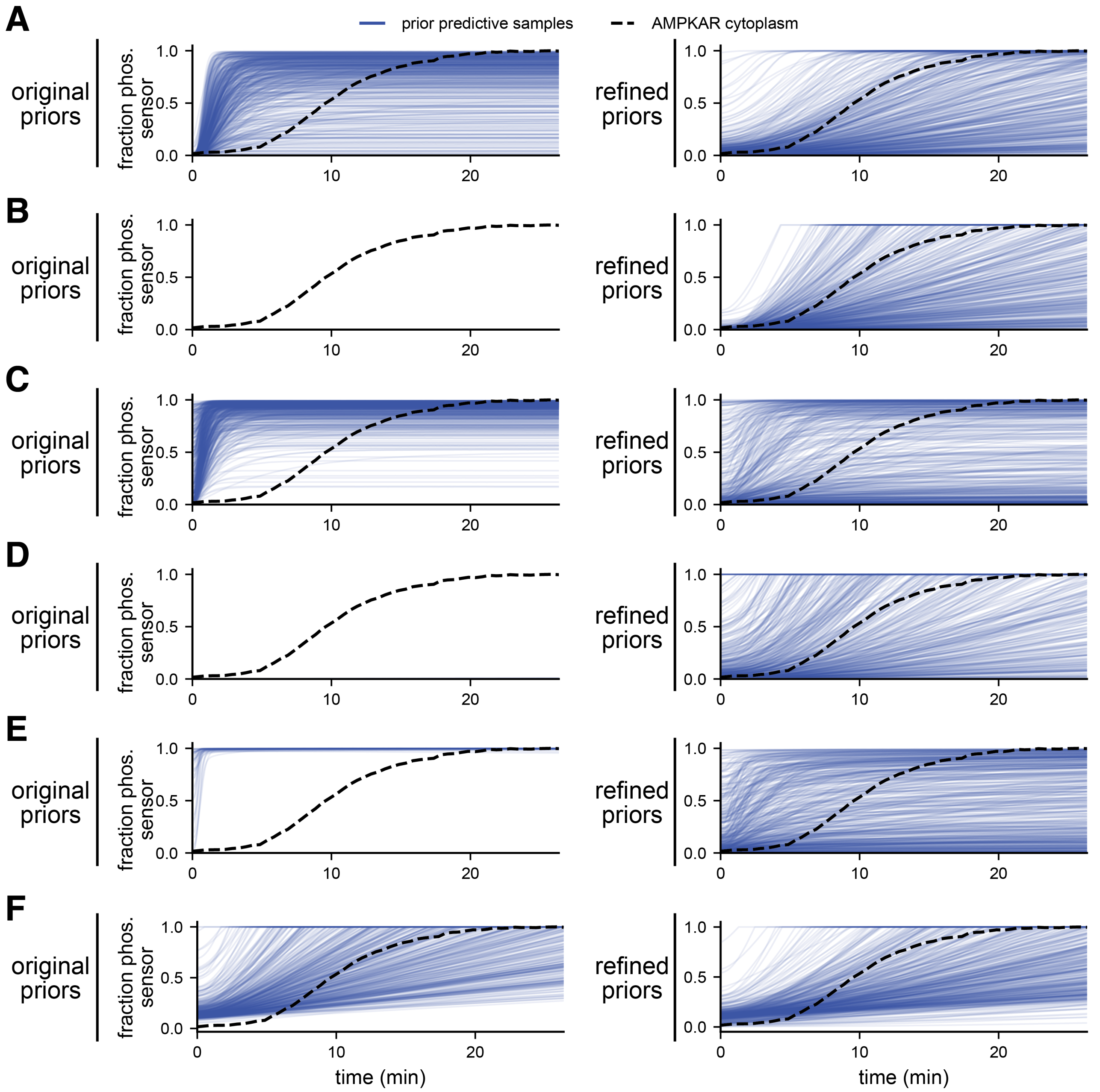

Supplementary Figure S2: **Prior predictive simulation samples with the original (left) and refined (right) priors for all models.** Dashed black line shows the data mean. Transparent blue lines show simulations with 400 prior samples.

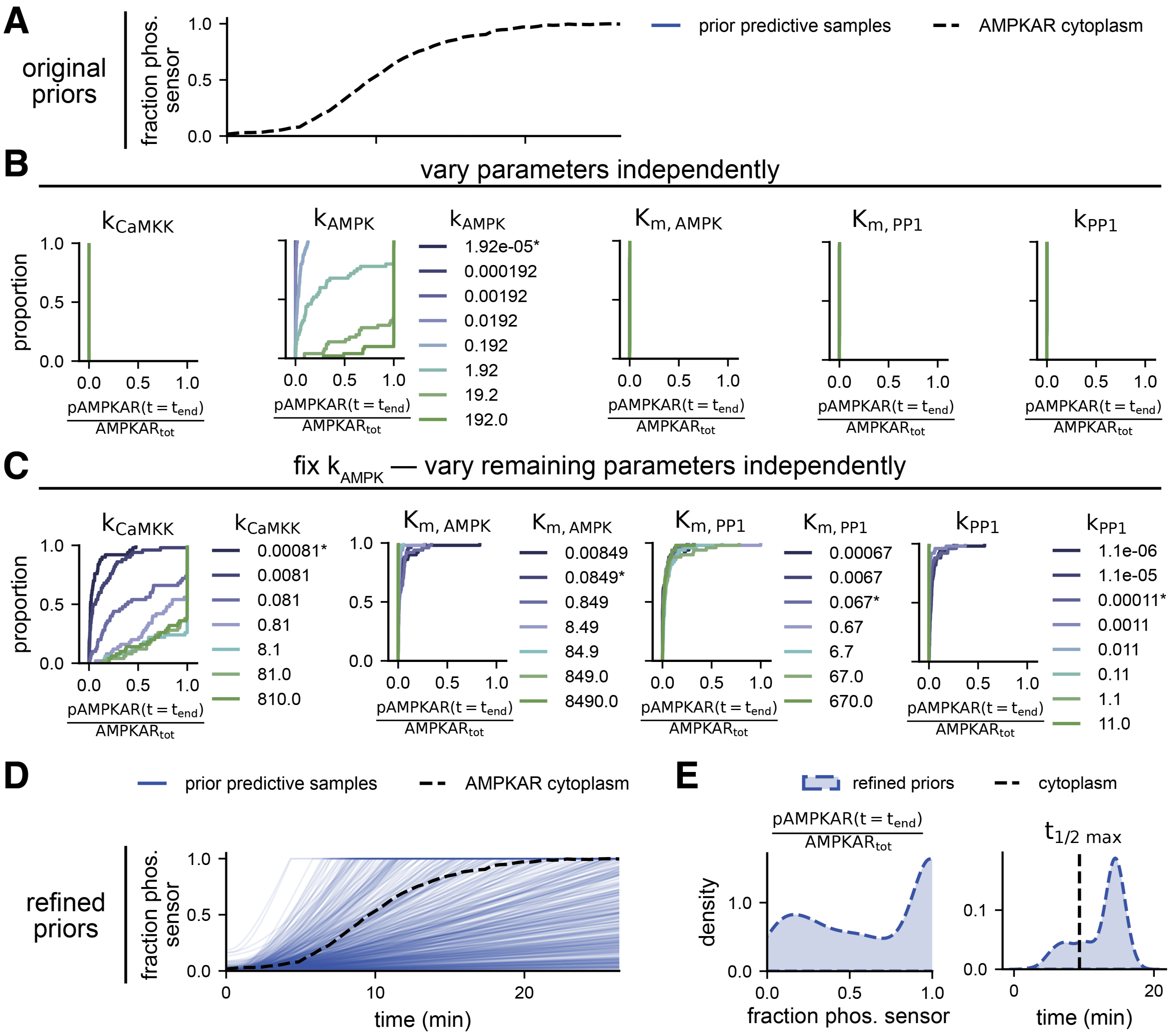

Supplementary Figure S3: **Prior refinement and nominal value selection for Model 2.** (**A**) Prior predictive distribution of predictions for priors based-on parameter ranges before any refinement. All simulations are nearly zero for all time. (**B**) Empirical cumulative density functions (CDF) for the final fraction of activated AMPKAR from prior predictive samples with single-parameter variations of nonidentifiable parameters. Only k_{AMPK} affects the strength of AMPKAR response. (**C**) Empirical CDFs for the final fraction of activated AMPKAR from prior predictive samples with single-parameter variations of nonidentifiable parameters with k_{AMPK} fixed to 0.192. Only k_{CaMKK} affects the strength of AMPKAR response. (**D**) Prior predictive distribution of predictions for refined priors and updated values for k_{AMPK} and k_{CaMKK}. (**E**) Estimated probability densities of key quantities after prior refinement.

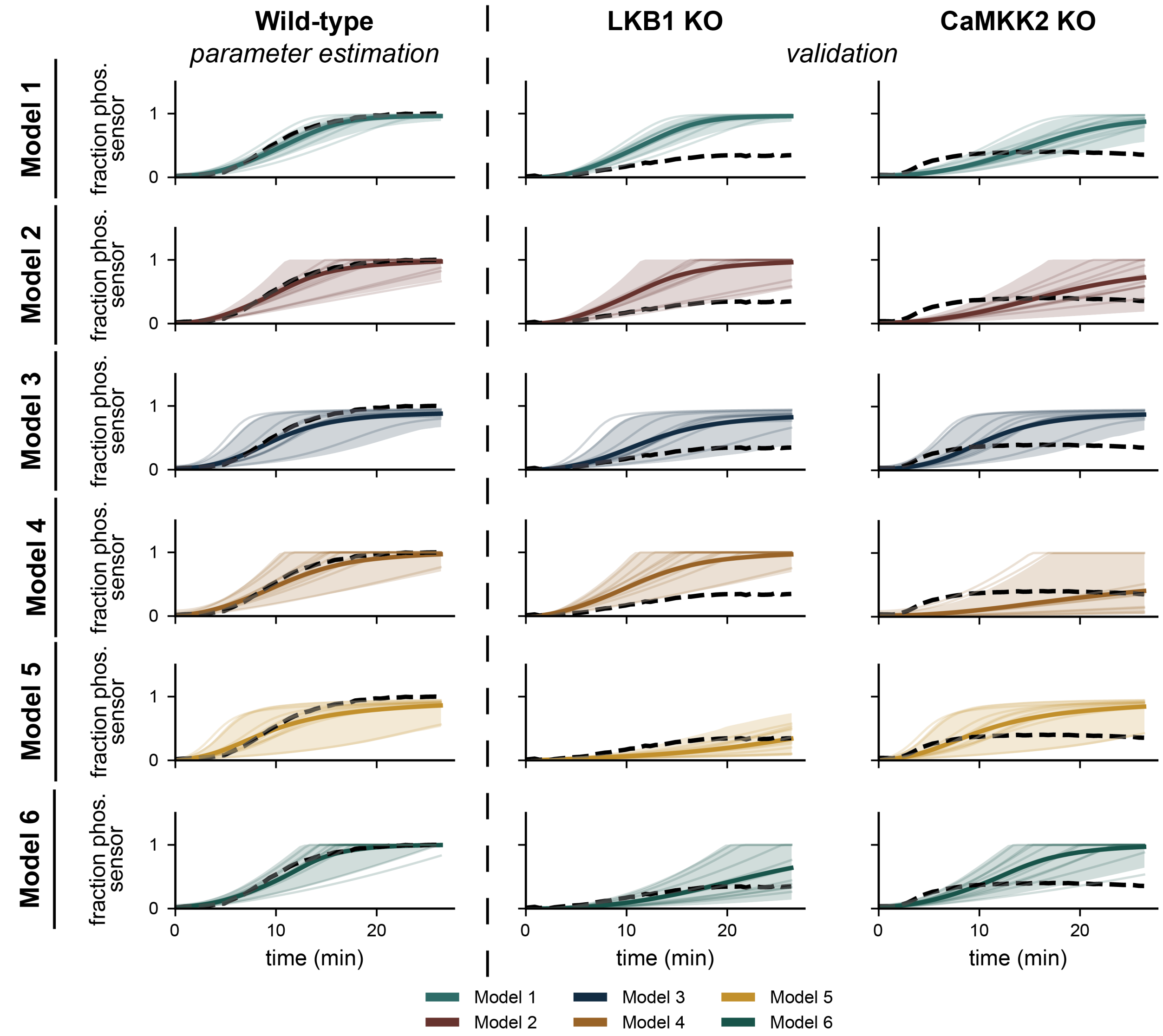

Supplementary Figure S4: **Posterior predictions for all models with parameters estimated with only wild-type data.** Dashed black lines show the data mean. Solid colored lines show the posterior mean, the shaded band shows the 95% credible interval, and the transparent lines show 10 samples from the posterior density. LKB1 and CaMKK2 knockout conditions are forward predictions.

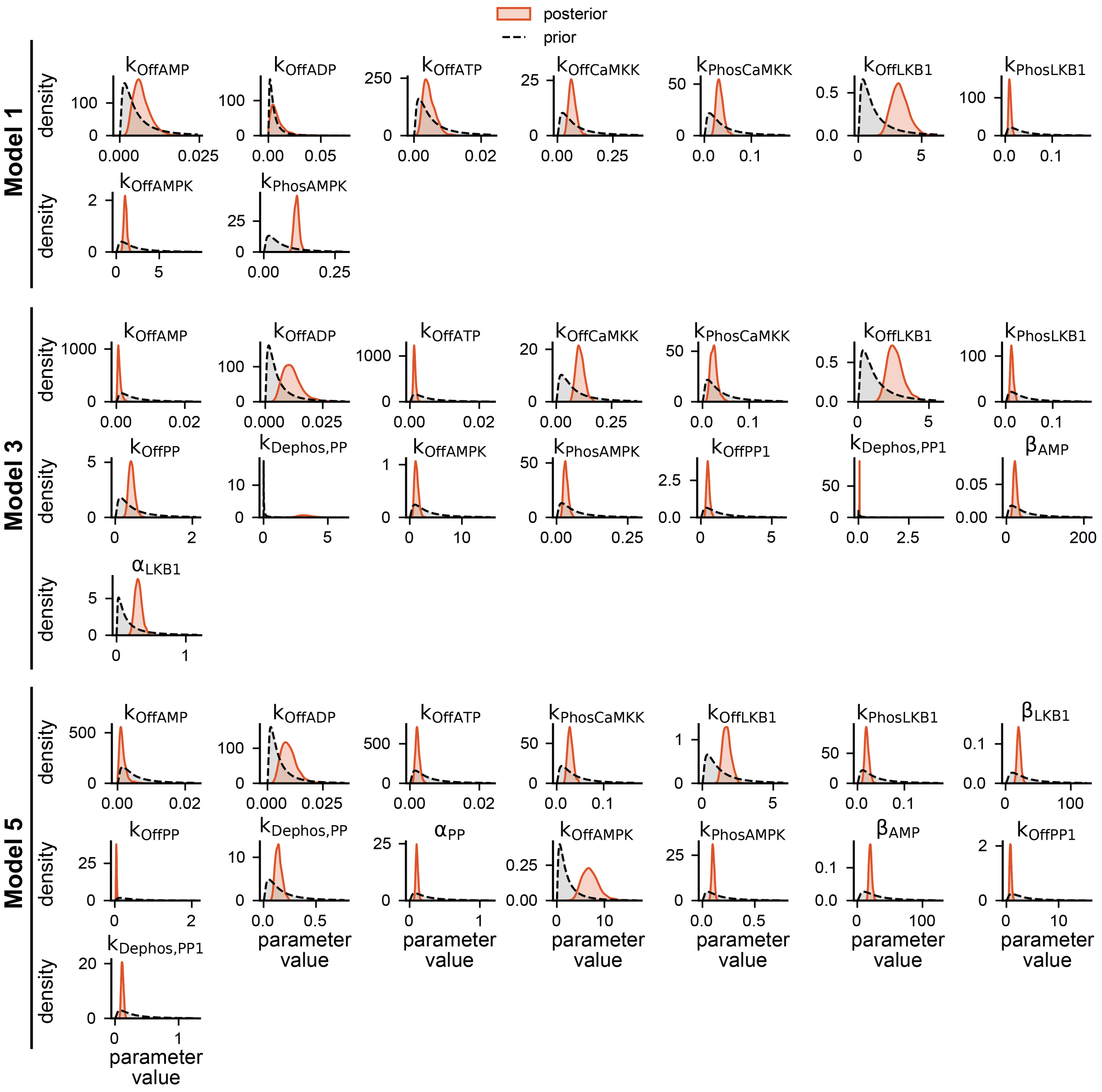

Supplementary Figure S5: **Estimated marginal posterior (orange) and prior (black dashed) densities of models with mass action kinetics, Models 1, 3, and 5.** Densities were estimated by fitting a kernel density estimator to 1000 posterior samples. Posterior was sampled using the Pathfinder variational inference algorithm [11].

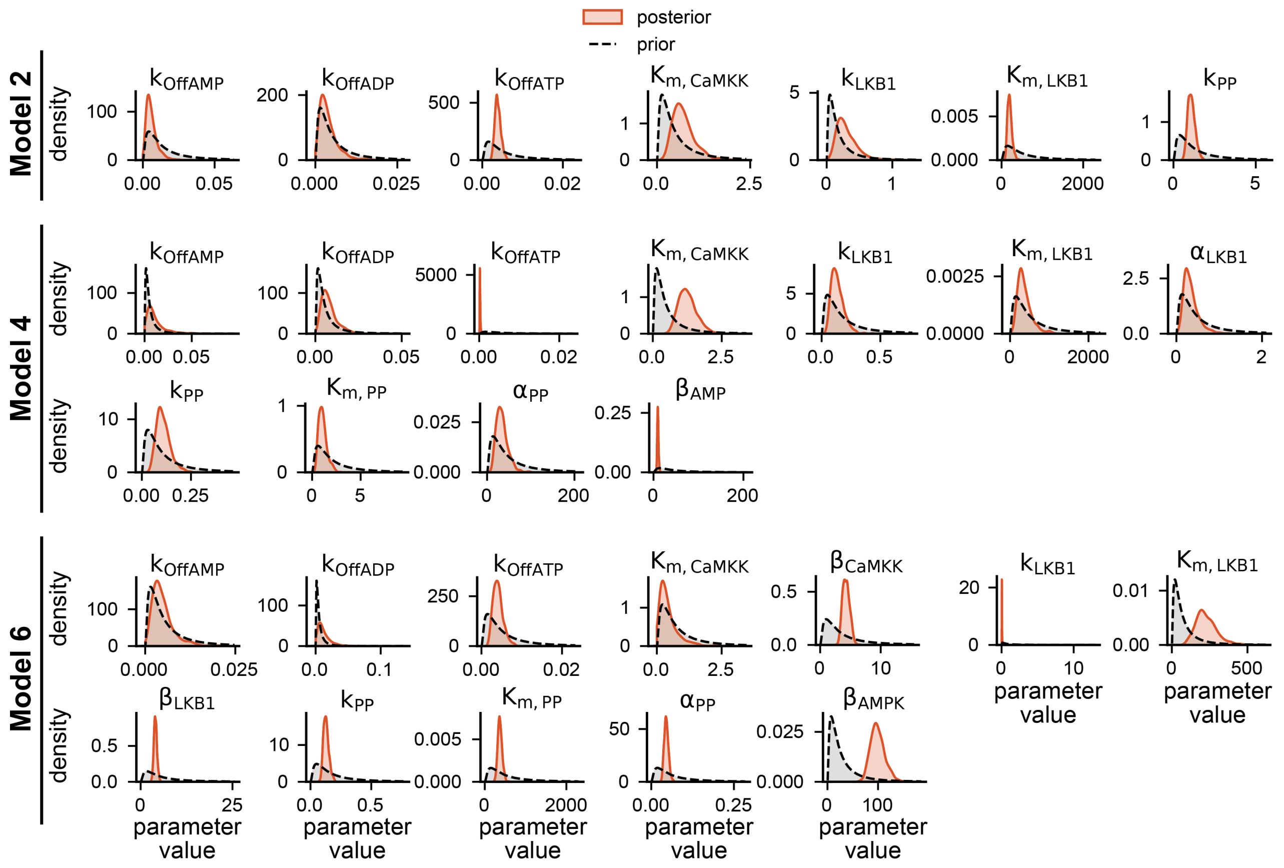

Supplementary Figure S6: **Estimated marginal posterior (orange) and prior (black dashed) densities of models with Michaelis-Menten kinetics, Models 2, 4, and 6.** Densities were estimated by fitting a kernel density estimator to 1000 posterior samples. Posterior was sampled using the Pathfinder variational inference algorithm [11].

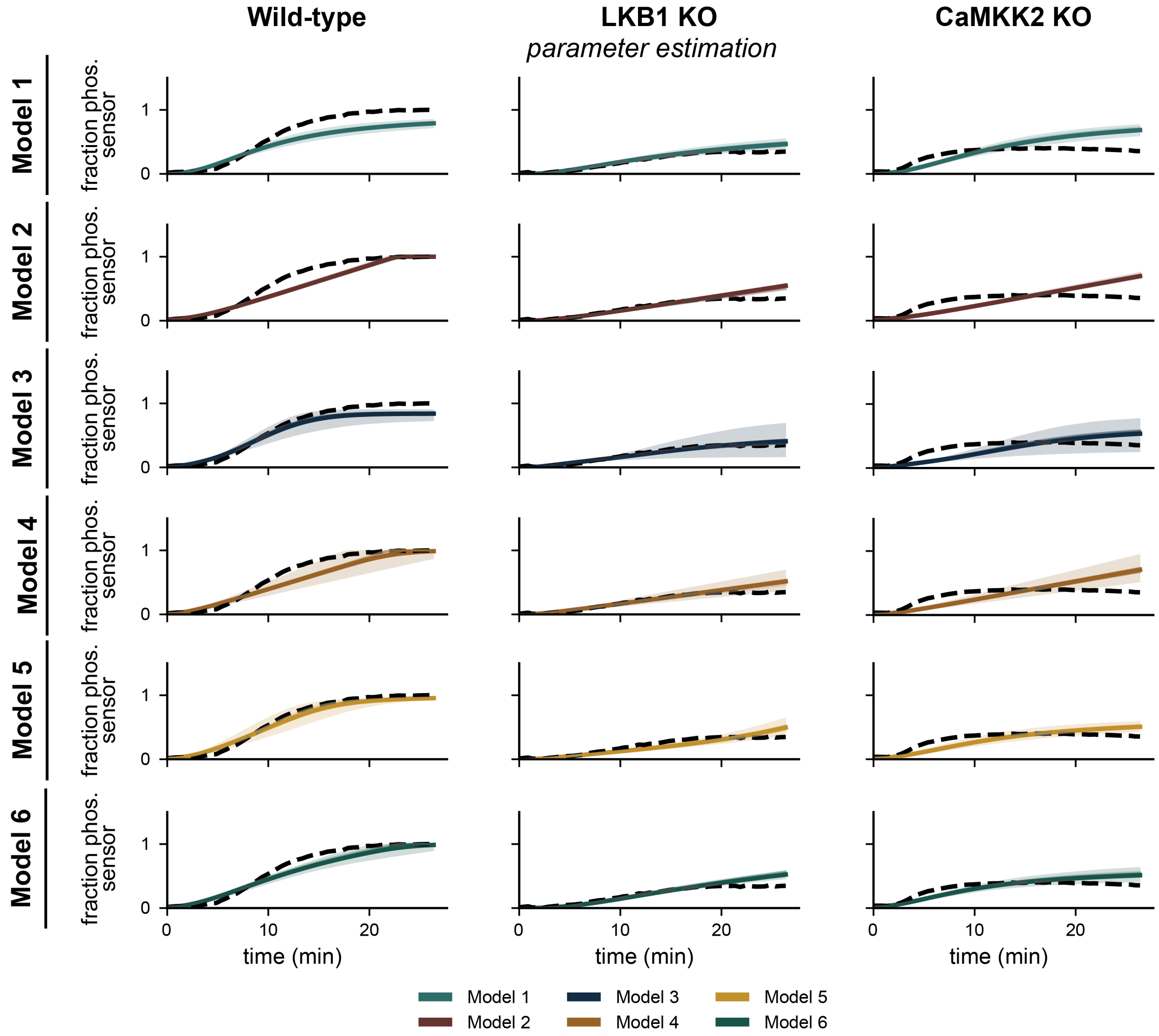

Supplementary Figure S7: **Posterior predictions for all models with parameters estimated with data from all three conditions simultaneously.** Dashed black lines show the data mean. Solid colored lines show the posterior mean, the shaded band shows the 95% credible interval, and the transparent lines show 10 samples from the posterior density.

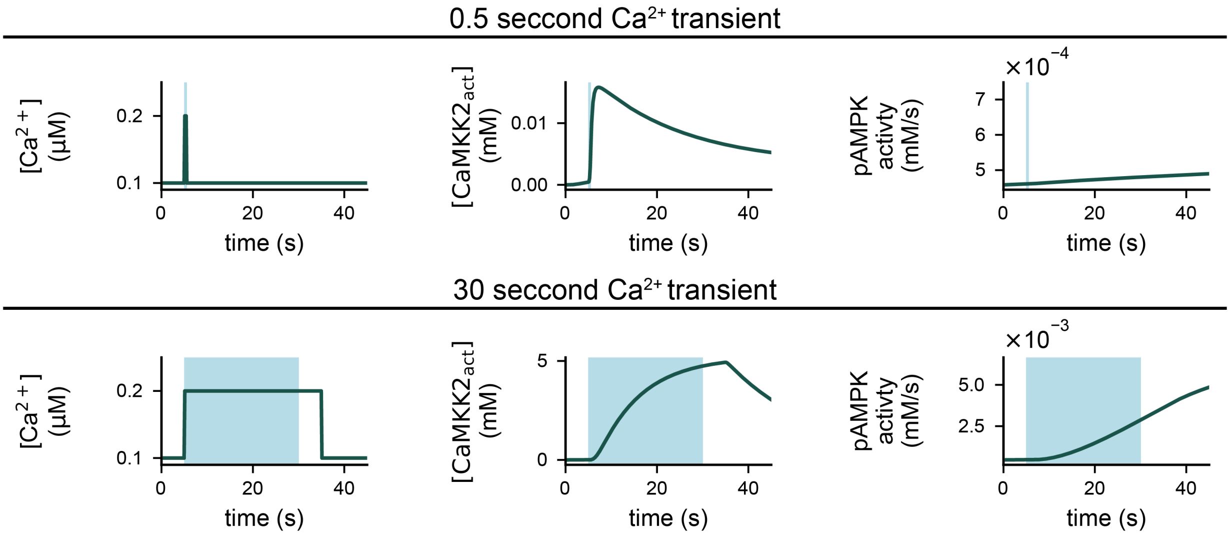

Supplementary Figure S8: **CaMKK2 responds transiently to transient calcium inputs.** Calcium is prescribed to vary transiently increasing from baseline and returning to baseline after 0.5 second (top) or 30 seconds (bottom). Black traces show predicted trajectories and blue shading indicates the time period when the stimulus is active.

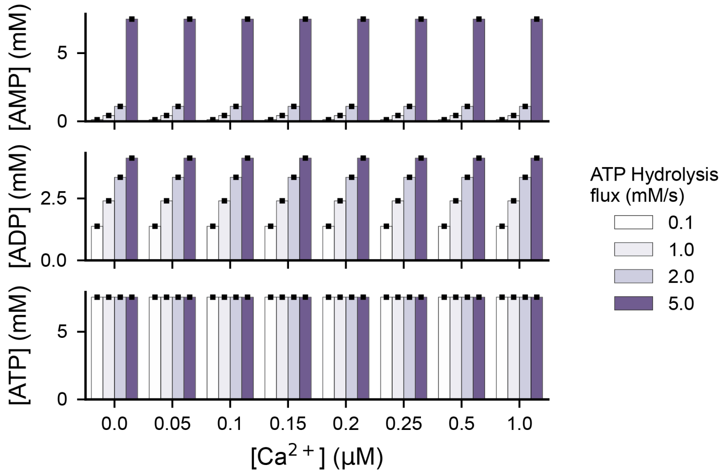

Supplementary Figure S9: **AMP, ADP and ATP concentrations during pulse inputs.** Corresponds to Figure 6B.

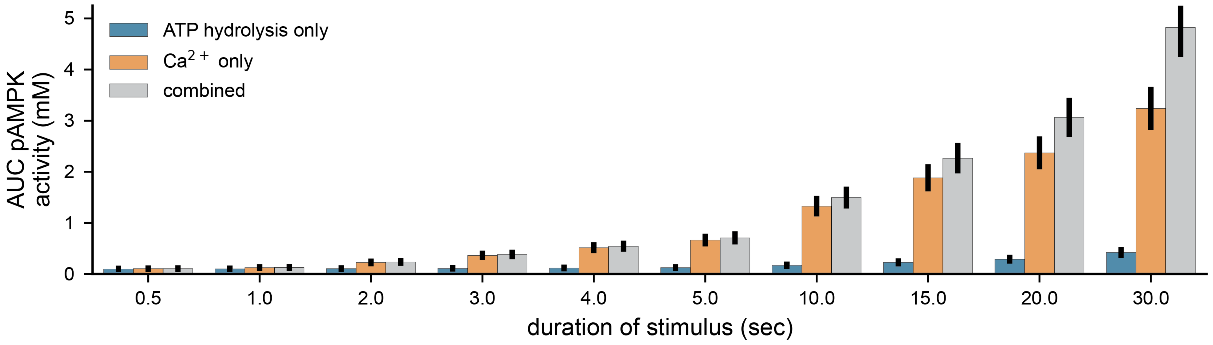

Supplementary Figure S10: **Area under the curve as a function of stimulus duration.** Stimuli strengths were either $[Ca^{2+}] = 0.1$ µM (Ca^{2+} only), ATP flux = 2.0 mM/s (ATP hydrolysis only), or both (combined).

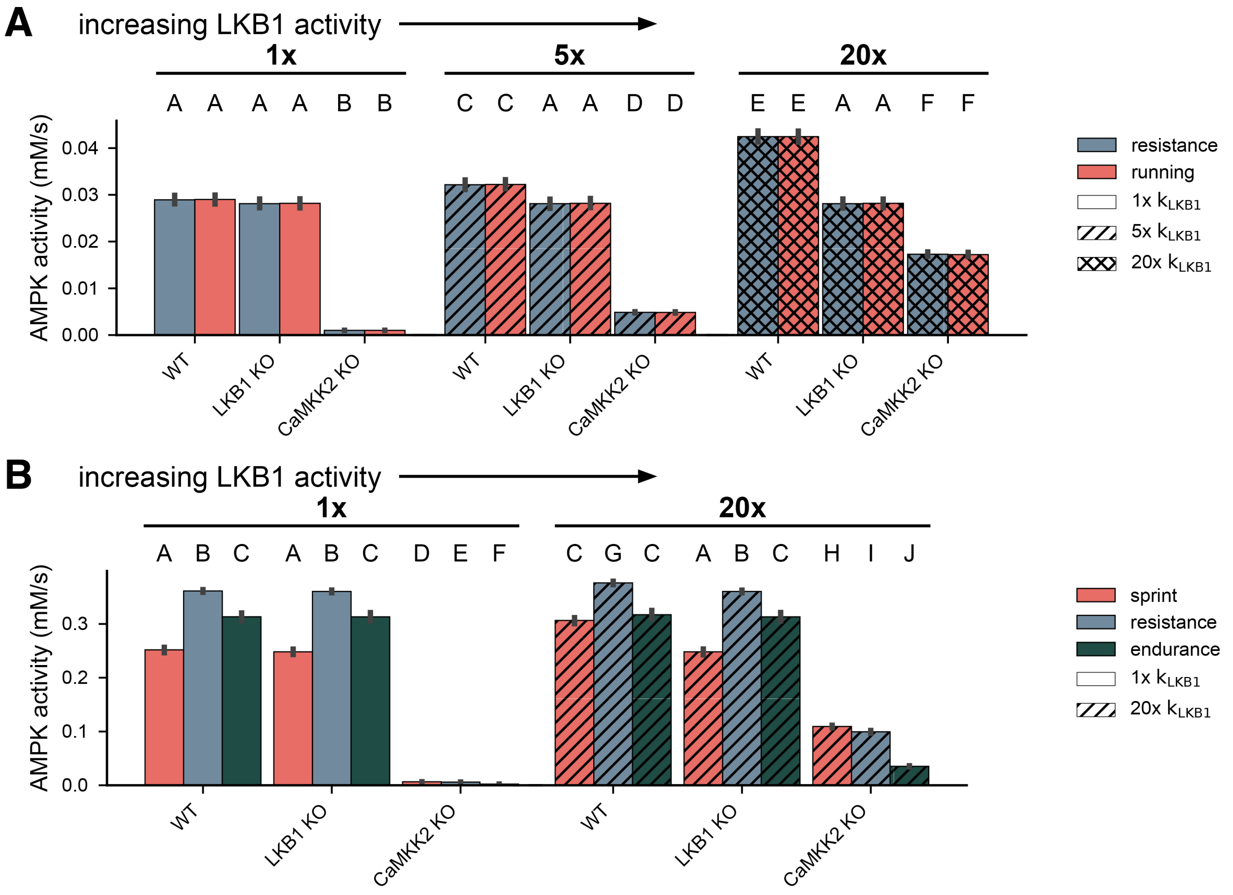

Supplementary Figure S11: **Effects of increased LKB1 activity on exercise-induced AMPK activity.** (**A**) Maximum pAMPK activity during short-term exercise for wild-type LKB1 KO, and CaMKK2 KO conditions with LKB1 that is five (5x) and twenty (20x) times more active. (**B**) Maximum pAMPK activity during long-term exercise for wild-type LKB1 KO, and CaMKK2 KO conditions with LKB1 that is twenty times (20x) more active. (A,B) Letters indicate statistical significance; groups with different letters are significantly different, and groups with like letters are significantly different. Significance was evaluated with a pairwise t-test and assessed at the level of 0.05 for n=50 independent samples in each group.

Supplementary Tables

Supplementary Table S1: **Prior parameters for the single enzyme-mediated reaction models shown in Figure 1**.

Model	Parameter	Units	Prior
Mass action	k_f	1/(mM s)	Gamma ($\alpha = 3.14, \beta = 33.01$)
Mass action	k_r	1/s	Gamma ($\alpha = 3.14, \beta = 132.04$)
Mass action	k_{cat}	1/s	Gamma ($\alpha = 3.14, \beta = 33.01$)
Michaelis-Menten	V_{max}	1/s	Gamma ($\alpha = 4.45, \beta = 420.00$)
Michaelis-Menten	K_m	mM	Gamma ($\alpha = 4.45, \beta = 1680.00$)
Hill-type	V_{max}	1/s	Gamma ($\alpha = 4.45, \beta = 4200.00$)
Hill-type	K_m	mM	Gamma ($\alpha = 3.14, \beta = 132.04$)
Hill-type	n	unitless	TruncatedNormal* ($\mu = 1.39, \sigma = 0.36, \text{lb} = 1, \text{ub} = \infty$)

* truncated normal distribution is truncated at one, so support is over $[1, \infty)$

Supplementary Table S2: **Identifiability of two-reaction models**. Naming indicates the kinetic formulation used for the first model and the second reaction, respectively. For example, mass action-mass action uses mass action kinetics for both reactions. Abbreviations: MA–mass action, MM–Michaelis-Menten, Hill–Hill-type.

**Model**	**Locally identifiable** $y_{\text{obs}} = [P]$	**NOT identifiable** $y_{\text{obs}} = [P]$	**Locally identifiable** $y_{\text{obs}} = \frac{[P]}{[S]_{\text{tot}}}$	**NOT identifiable** $y_{\text{obs}} = \frac{[P]}{[S]_{\text{tot}}}$
MA–MA	$k_{f,1}$, $k_{r,1}$, $k_{\text{cat},1}$,$k_{f,2}$, $k_{r,2}$, $k_{\text{cat},2}$	–	$k_{r,1}$, $k_{\text{cat},1}$, $k_{r,2}$, $k_{\text{cat},2}$	$k_{f,1}$, $k_{f,2}$
MA–MM	$k_{f,1}$, $k_{r,1}$, $k_{\text{cat},1}$, $V_{\text{max},2}$, $K_{m,2}$	–	$k_{r,1}$, $k_{\text{cat},1}$	$k_{f,1}$, $V_{\text{max},2}$, $K_{m,2}$
MA–Hill	$k_{f,1}$, $k_{r,1}$, $k_{\text{cat},1}$, $V_{\text{max},2}$, $K_{m,2}$	–	$k_{r,1}$, $k_{\text{cat},1}$	$k_{f,1}$, $V_{\text{max},2}$, $K_{m,2}$
MM–MM	$V_{\text{max},1}$, $K_{m,1}$, $V_{\text{max},2}$, $K_{m,2}$	–	–	$V_{\text{max},1}$, $K_{m,1}$, $V_{\text{max},2}$, $K_{m,2}$
MM–MA	$V_{\text{max},1}$, $K_{m,1}$, $k_{f,2}$, $k_{r,2}$, $k_{\text{cat},2}$	–	$V_{\text{max},1}$, $K_{m,1}$, $k_{f,2}$, $k_{r,2}$, $k_{\text{cat},2}$	–
MM–Hill	$V_{\text{max},1}$, $K_{m,1}$, $V_{\text{max},2}$, $K_{m,2}$	–	–	$V_{\text{max},1}$, $K_{m,1}$, $V_{\text{max},2}$, $K_{m,2}$
Hill–Hill	$V_{\text{max},1}$, $K_{m,1}$, $V_{\text{max},2}$, $K_{m,2}$	–	–	$V_{\text{max},1}$, $K_{m,1}$, $V_{\text{max},2}$, $K_{m,2}$
Hill–MA	$V_{\text{max},1}$, $K_{m,1}$, $k_{f,2}$, $k_{r,2}$, $k_{\text{cat},2}$	–	$k_{r,2}$, $k_{\text{cat},2}$	$V_{\text{max},1}$, $K_{m,1}$, $k_{f,2}$
Hill–MM	$V_{\text{max},1}$, $K_{m,1}$, $V_{\text{max},2}$, $K_{m,2}$	–	–	$V_{\text{max},1}$, $K_{m,1}$, $V_{\text{max},2}$, $K_{m,2}$

Supplementary Table S3: **Fluxes for the metabolism module (Module 1)**.

**Reaction**	**Flux**	**Reference**
Oxidative phos.	$J_{\text{OxPhos}} = \frac{V_{\text{max,OxPhos}}\left(\frac{[ADP]}{K_{ADP}}\right)^n}{1+\left(\frac{[ADP]}{K_{ADP}}\right)^n}$	[3]
Glycolysis	$J_{\text{glycolysis}} = k_{\text{glycolysis}}[\text{ADP}]$	n/a
ATP Hydrolysis	$J_{\text{hydro}} = k_{\text{hydro}}[\text{ATP}]$	[1]
Adenylate kinase (forward)	$J_{\text{AK,for}} = \frac{\frac{V_{\text{AK,for}}[\text{ATP}][\text{AMP}]}{\text{kmt}\times\text{kmm}}}{1+\frac{[\text{ATP}]}{\text{kmt}}+\frac{[\text{AMP}]}{\text{kmm}}+\frac{[\text{ATP}][\text{AMP}]}{\text{kmt}\times\text{kmm}}+\frac{2[\text{ADP}]}{\text{kmd}}+\frac{[\text{ADP}]^2}{\text{kmd}^2}}$	[4]
Adenylate kinase (reverse)*	$J_{\text{AK,rev}} = \frac{\frac{V_{\text{AK,rev}}[\text{ADP}]^2}{\text{kmd}^2}}{1+\frac{[\text{ATP}]}{\text{kmt}}+\frac{[\text{AMP}]}{\text{kmm}}+\frac{[\text{ATP}][\text{AMP}]}{\text{kmt}\times\text{kmm}}+\frac{2[\text{ADP}]}{\text{kmd}}+\frac{[\text{ADP}]^2}{\text{kmd}^2}}$	[4]
Adenylate kinase	$J_{\text{AK}} = J_{\text{AK,for}} - J_{\text{AK,rev}}$	[4]
Creatine kinase (forward)	$J_{\text{CK,for}} = \frac{\frac{V_{\text{CK,for}}K_{iq}K_p[\text{ADP}][\text{PCr}]}{K_{ia}K_b}}{1+\frac{[\text{ADP}]}{K_{ia}}+\frac{[\text{PCr}]}{K_{ib}}+\frac{[\text{ATP}]}{K_{iq}}+\frac{[\text{ADP}][\text{PCr}]}{K_{ia}K_b}+\frac{([\text{Cr}]_{\text{total}}-[\text{PCr}])[\text{ATP}]}{K_{iq}K_p}}$	[4]
Creatine kinase (reverse)**	$J_{\text{CK,rev}} = \frac{\frac{V_{\text{CK,rev}}([\text{Cr}]_{\text{total}}-[\text{PCr}])[\text{ATP}]}{K_{iq}K_p}}{1+\frac{[\text{ADP}]}{K_{ia}}+\frac{[\text{PCr}]}{K_{ib}}+\frac{[\text{ATP}]}{K_{iq}}+\frac{[\text{ADP}][\text{PCr}]}{K_{ia}K_b}+\frac{([\text{Cr}]_{\text{total}}-[\text{PCr}])[\text{ATP}]}{K_{iq}K_p}}$	[4]
Creatine kinase	$J_{\text{CK}} = J_{\text{CK,rev}} - J_{\text{CK,for}}$	[4]

* $V_{\text{AK,rev}} \coloneqq \frac{V_{\text{AK,for}}\times\text{kmd}^2}{K_{\text{eqAK}}\times\text{kmt}\times\text{kmm}}$

** $V_{\text{CK,rev}} \coloneqq \frac{V_{\text{CK,for}}K_{iq}K_p}{K_{\text{eqCK}}K_{ia}K_b}$

Supplementary Table S4: **Model parameters for metabolism module (Module 1).**

**Reaction**	**Parameter**	**Units**	**Nominal value**	**Reference/ Rationale**
Oxidative phos.	$V_{\mathrm{max,OxPhos}}$	mM/s	0.5	[1, 3]
	K_{ADP}	mM	5.8×10^{-2}	[1, 3]
	n	unitless	2.568	[1, 3]
Glycolysis	k_{Gly}	mM/s	0.5	derived*
ATP Hydrolysis	k_{hydro}	1/s	1.5×10^{-1}	tuned
Adenylate kinase	$V_{\mathrm{AK,for}}$	mM/s	14.66	[1, 4]
	kmt	mM	0.27	[1, 4]
	kmm	mM	0.32	[1, 4]
	kmd	mM	0.35	[1, 4]
	K_{eqAK}	unitless	2.221	[1, 4]
Creatine kinase	$V_{\mathrm{CK,for}}$	mM/s	1×10^{2}	[1, 3]
	K_b	mM	1.11	[1, 3]
	K_{ia}	mM	0.135	[1, 3]
	K_{ib}	mM	3.9	[1, 3]
	K_{iq}	mM	3.5	[1, 3]
	K_p	mM	3.8	[1, 3]
	K_{eqCK}	unitless	$1.77 \times 10^{(9-\mathrm{pH})}$	assume pH = 7 [1, 3]
	$[\mathrm{Cr}]_{\mathrm{total}}$	mM	39.0	[1, 3]

* See note in Supplementary Section 2.1.

Supplementary Table S5: **AMPK model parameters**. Nominal values and bounds.

Parameter	units	nominal value	bounds
k_{OnAMP}	1/(mM × s)	1.0	–
k_{OffAMP}	1/s	2.5±0.6 [6][a]	[2.5e-9, 25]
k_{OnADP}	1/(mM × s)	1.0	–
k_{OffADP}	1/s	1.5±0.4 [6]	[1.5e-9, 15]
k_{OnATP}	1/(mM × s)	1.0	–
k_{OffATP}	1/s	1.7±0.5 [6]	[1.7e-9, 17]
$k_{\text{on,LKB1}}$	1/(mM × s)	1.0	–
$k_{\text{off,LKB1}}$	1/s	approx. from Mich. Ment.	[0.001396, 1396.0]
$k_{\text{phos,LKB1}}$	1/s	3.92×10^{-2}	[3.92e-06, 0.392]
k_{LKB1}	1/s	$3.92 \times 10^{-2} \pm 3.4 \times 10^{-3}$ [1, 12]**	[3.92e-06, 0.392]
$K_{\text{m,LKB1}}$	mM	1.4±0.24 [1, 12][b]	[0.00139992, 1,396.392]
α_{LKB1}	–	–	[1e-4, 1.0]
β_{LKB1}	–	–	[0.95, 113.03]
$k_{\text{on,CaMKK}}$	1/(mM × s)	1.0	–
$k_{\text{off,CaMKK}}$	1/s	approx. from Mich. Ment.	[1.11e-05, 1.11]
$k_{\text{phos,CaMKK}}$	1/s	3.92e-2[c]	[3.92e-05, 0.392]
k_{CaMKK}	1/s	3.92e-2[c]	[3.92e-05, 0.392]
$K_{\text{m,CaMKK}}$	μM	15	[5.03e-5, 1.502]
β_{CaMKK}	–	–	[0.95, 113.03]
$k_{\text{on,PP}}$	1/(mM × s)	1.0	–
$k_{\text{off,PP}}$	1/s	approx. from Mich. Ment.	[5.6e-06, 5.6]
$k_{\text{dephos,PP}}$	1/s	1.1e-1[d]	[1.1e-06, 0.11]
k_{PP}	1/s	$1.1 \times 10^{-1} \pm 4.0 \times 10^{-3}$ [1, 13][d]	[1.1e-06, 0.11]
$K_{\text{m,PP}}$	mM	$6.7 \times 10^{-2} \pm 1.3 \times 10^{-2}$ [1, 13]	[6.7e-6, 5.71]
α_{PP}	–	–	[1.0, 100.0]
$k_{\text{on,PP1}}$	1/(mM × s)	1.0	–
$k_{\text{off,PP1}}$	1/s	approx. from Mich. Ment.[e]	[5.6e-06, 5.6]
$k_{\text{dephos,PP1}}$	1/s	1.1e-1[e]	[1.1e-06, 0.11]
k_{PP1}	1/s	[e]	[1.1e-06, 0.11]
$K_{\text{m,PP1}}$	mM	[e]	[6.7e-6, 5.71]
$k_{\text{on,AMPK}}$	1/(mM × s)	1.0	–
$k_{\text{off,AMPK}}$	1/s	approx. from Mich. Ment.	[0.000301, 301.0]
$k_{\text{phos,AMPK}}$	1/s	6.33 ± 0.29 [14][f]	[0.00633, 6330.0]
k_{AMPK}	1/s	6.33 ± 0.29 [14]	[0.00633, 6330.0]
$K_{\text{m,AMPK}}$	μM	4.67 ± 1.48 [14][f]	[0.006631, 6,631]
β_{AMPK}	–	–	[1.0, 100.0]
β_{AMP}	–	–	[0.95, 113.03]

[a] K_d for strong binding site from [6]

[b] Set to $V_{\max}$ (assume enzyme concentration of 1.0 mM). Originally from [12]. Converted by [1] using unknown conversion factors.

[c] Assume same as LKB1.

[d] Set to $V_{\max}$ (assume enzyme concentration of 1.0 mM). The original nominal value had units of μM/min/mg [13]. The conversion was done by [1] using unknown conversion factors.

[e] Same as PP.

[f] Values based on Table 1 of [14].

Supplementary Table S6: **Local identifiability.**

Model	Locally Identifiable	NOT Locally Identifiable
1	kOnAMP, kOffAMP, kOnADP, kOffADP, kOnATP, kOffATP, kOnCaMKK, kOffCaMKK, kPhosCaMKK, kOnLKB1, kOffLKB1, kPhosLKB1, kOnPP, kOffPP, kDephosPP, kOnAMPK, kOffAMPK, kPhosAMPK, kOnPP1, kOffPP1, kDephosPP1	
2	kOnAMP, kOffAMP, kOnADP, kOffADP, kOnATP, kOffATP, KmCaMKK, kLKB1, KmLKB1, kPP, KmPP	kCaMKK, kAMPK, KmAMPK, kPP1, KmPP1
3	kOnAMP, kOffAMP, kOnADP, kOffADP, kOnATP, kOffATP, kOnCaMKK, kOffCaMKK, kPhosCaMKK, kOnLKB1, kOffLKB1, alphaLKB1, kPhosLKB1, kOnPP, kOffPP, alphaPP, kDephosPP, kOnAMPK, kOffAMPK, kPhosAMPK, betaAMP, kOnPP1, kOffPP1, kDephosPP1	
4	kOnAMP, kOffAMP, kOnADP, kOffADP, kOnATP, kOffATP, KmCaMKK, kLKB1, KmLKB1, alphaLKB1, kPP, KmPP, alphaPP, betaAMP	kCaMKK, kAMPK, KmAMPK, kPP1, KmPP1
5	kOnAMP, kOffAMP, kOnADP, kOffADP, kOnATP, kOffATP, kOnCaMKK, kOffCaMKK, kPhosCaMKK, kOnLKB1, kOffLKB1, kPhosLKB1, kOnPP, kOffPP, kDephosPP, kOnAMPK, kOffAMPK, kPhosAMPK, kOnPP1, kOffPP1, kDephosPP1, alphaPP, betaAMP, betaCaMKK, betaLKB1	
6	kOnAMP, kOffAMP, kOnADP, kOffADP, kOnATP, kOffATP, KmCaMKK, kLKB1, KmLKB1, kPP, KmPP, betaAMPK, betaLKB1, betaCaMKK, alphaPP	kCaMKK, kAMPK, KmAMPK, kPP1, KmPP1

Supplementary Table S7: **Sobol total sensitivity indices for Model 1.** Bold values indicate influential parameters with a mean index greater than or equal to 0.01.

		max fraction act. AMPKAR			Time to half-max		
**Parameter**	**Function**	**WT**	**LKB1 KO**	**CaMKK2 KO**	**WT**	**LKB1 KO**	**CaMKK2 KO**
k_{OffAMP}	AMP binding	**0.135 ± 0.043**	**0.151 ± 0.044**	**0.151 ± 0.048**	**0.067 ± 0.017**	**0.056 ± 0.012**	**0.073 ± 0.015**
k_{OffADP}	ADP binding	**0.087 ± 0.035**	**0.082 ± 0.034**	**0.085 ± 0.037**	**0.059 ± 0.018**	**0.095 ± 0.033**	**0.056 ± 0.018**
k_{OffATP}	ATP binding	**0.219 ± 0.071**	**0.207 ± 0.059**	**0.184 ± 0.062**	**0.407 ± 0.118**	**0.342 ± 0.095**	**0.359 ± 0.098**
k_{OffCaMKK}	NaN	0.001 ± 0.001	**0.010 ± 0.004**	0.000 ± 0.000	0.001 ± 0.001	**0.019 ± 0.007**	0.000 ± 0.000
$k_{\mathrm{PhosCaMKK}}$	AMPK phos.	0.003 ± 0.003	**0.084 ± 0.045**	0.000 ± 0.000	0.004 ± 0.002	**0.155 ± 0.051**	0.000 ± 0.000
k_{OffLKB1}	AMPK phos.	0.001 ± 0.001	0.000 ± 0.000	0.007 ± 0.003	0.005 ± 0.001	0.000 ± 0.000	**0.023 ± 0.010**
k_{PhosLKB1}	AMPK phos.	0.004 ± 0.003	0.000 ± 0.000	**0.076 ± 0.044**	**0.021 ± 0.008**	0.000 ± 0.000	**0.126 ± 0.057**
k_{OffPP}	AMPK dephos.	0.004 ± 0.003	0.005 ± 0.003	0.002 ± 0.002	0.002 ± 0.001	0.004 ± 0.002	0.000 ± 0.000
k_{DephosPP}	AMPK dephos.	0.003 ± 0.003	0.003 ± 0.002	0.001 ± 0.001	0.001 ± 0.001	0.001 ± 0.001	0.001 ± 0.000
k_{OffAMPK}	AMPK kinase act.	**0.183 ± 0.055**	**0.166 ± 0.050**	**0.177 ± 0.053**	**0.108 ± 0.023**	**0.085 ± 0.020**	**0.106 ± 0.028**
k_{PhosAMPK}	AMPK kinase act.	**0.516 ± 0.108**	**0.454 ± 0.084**	**0.480 ± 0.096**	**0.545 ± 0.120**	**0.514 ± 0.122**	**0.493 ± 0.101**
k_{OffPP1}	AMKPAR dephos.	0.002 ± 0.001	0.002 ± 0.001	0.002 ± 0.001	0.003 ± 0.003	0.003 ± 0.003	0.002 ± 0.001
$k_{\mathrm{Dephos,PP1}}$	AMKPAR dephos.	0.007 ± 0.002	0.006 ± 0.001	0.007 ± 0.002	0.003 ± 0.001	0.004 ± 0.002	0.003 ± 0.001

Supplementary Table S8: **Sobol total sensitivity indices for Model 2.** Bold values indicate influential parameters with a mean index greater than or equal to 0.01.

		**max fraction act. AMPKAR**			**Time to half-max**		
**Parameter**	**Function**	**WT**	**LKB1 KO**	**CaMKK2 KO**	**WT**	**LKB1 KO**	**CaMKK2 KO**
k_{OffAMP}	AMP binding	**0.498 ± 0.089**	**0.509 ± 0.098**	**0.724 ± 0.158**	**0.806 ± 0.135**	**0.776 ± 0.131**	**0.478 ± 0.144**
k_{OffADP}	ADP binding	**0.174 ± 0.063**	**0.163 ± 0.054**	**0.038 ± 0.022**	**0.144 ± 0.056**	**0.158 ± 0.062**	**0.218 ± 0.091**
k_{OffATP}	ATP binding	**0.327 ± 0.086**	**0.310 ± 0.081**	**0.155 ± 0.076**	**0.255 ± 0.085**	**0.237 ± 0.088**	**0.242 ± 0.115**
$K_{m,\text{CaMKK}}$	AMPK phos.	**0.127 ± 0.043**	**0.147 ± 0.046**	0.000 ± 0.000	**0.149 ± 0.048**	**0.197 ± 0.068**	0.000 ± 0.000
k_{LKB1}	AMPK phos.	0.004 ± 0.003	0.000 ± 0.000	**0.215 ± 0.114**	0.007 ± 0.004	0.000 ± 0.000	**0.260 ± 0.093**
$K_{\text{m,LKB1}}$	AMPK phos.	**0.011 ± 0.005**	0.000 ± 0.000	**0.233 ± 0.079**	**0.018 ± 0.011**	0.000 ± 0.000	**0.557 ± 0.286**
k_{PP}	AMPK dephos.	**0.035 ± 0.016**	**0.037 ± 0.015**	**0.012 ± 0.007**	**0.017 ± 0.009**	**0.020 ± 0.011**	**0.085 ± 0.044**
$K_{\text{m,PP}}$	AMPK dephos.	**0.024 ± 0.009**	**0.024 ± 0.010**	0.009 ± 0.005	**0.022 ± 0.011**	**0.025 ± 0.014**	**0.036 ± 0.018**

Supplementary Table S9: **Sobol total sensitivity indices for Model 3.** Bold values indicate influential parameters with a mean index greater than or equal to 0.01.

		**max fraction act. AMPKAR**			**Time to half-max**		
**Parameter**	**Function**	**WT**	**LKB1 KO**	**CaMKK2 KO**	**WT**	**LKB1 KO**	**CaMKK2 KO**
k_{OffAMP}	AMP binding	**0.198 ± 0.053**	**0.227 ± 0.064**	**0.208 ± 0.055**	**0.416 ± 0.108**	**0.454 ± 0.133**	**0.415 ± 0.125**
k_{OffADP}	ADP binding	**0.086 ± 0.038**	**0.072 ± 0.040**	**0.081 ± 0.031**	**0.057 ± 0.024**	**0.028 ± 0.012**	**0.063 ± 0.031**
k_{OffATP}	ATP binding	**0.075 ± 0.036**	**0.082 ± 0.046**	**0.074 ± 0.032**	**0.584 ± 0.210**	**0.348 ± 0.145**	**0.517 ± 0.152**
k_{OffCaMKK}	NaN	0.001 ± 0.000	0.007 ± 0.004	0.000 ± 0.000	0.002 ± 0.001	**0.023 ± 0.013**	0.000 ± 0.000
$k_{\mathrm{PhosCaMKK}}$	AMPK phos.	0.002 ± 0.001	**0.055 ± 0.026**	0.000 ± 0.000	**0.025 ± 0.027**	**0.248 ± 0.102**	0.000 ± 0.000
k_{OffLKB1}	AMPK phos.	0.002 ± 0.001	0.000 ± 0.000	0.007 ± 0.005	0.010 ± 0.004	0.000 ± 0.000	**0.047 ± 0.052**
k_{PhosLKB1}	AMPK phos.	0.007 ± 0.004	0.000 ± 0.000	**0.026 ± 0.015**	**0.045 ± 0.023**	0.000 ± 0.000	**0.150 ± 0.087**
k_{OffPP}	AMPK dephos.	0.002 ± 0.002	0.004 ± 0.002	0.002 ± 0.002	0.005 ± 0.005	**0.016 ± 0.014**	0.005 ± 0.005
k_{DephosPP}	AMPK dephos.	0.001 ± 0.000	0.003 ± 0.002	0.001 ± 0.000	0.002 ± 0.002	**0.011 ± 0.010**	0.002 ± 0.002
k_{OffAMPK}	AMPK kinase act.	**0.036 ± 0.012**	**0.036 ± 0.015**	**0.036 ± 0.013**	**0.052 ± 0.037**	**0.081 ± 0.065**	**0.044 ± 0.029**
k_{PhosAMPK}	AMPK kinase act.	**0.135 ± 0.070**	**0.123 ± 0.075**	**0.134 ± 0.071**	**0.036 ± 0.028**	**0.020 ± 0.014**	**0.034 ± 0.026**
k_{OffPP1}	AMKPAR dephos.	**0.030 ± 0.008**	**0.029 ± 0.010**	**0.031 ± 0.011**	**0.022 ± 0.012**	**0.014 ± 0.009**	**0.023 ± 0.013**
$k_{\mathrm{Dephos,PP1}}$	AMKPAR dephos.	**0.501 ± 0.109**	**0.471 ± 0.128**	**0.508 ± 0.107**	**0.087 ± 0.054**	**0.057 ± 0.048**	**0.072 ± 0.037**
α_{LKB1}	AMPK phos.	0.001 ± 0.000	0.000 ± 0.000	0.004 ± 0.004	0.004 ± 0.002	0.000 ± 0.000	**0.015 ± 0.009**
α_{PP}	AMPK dephos.	0.002 ± 0.001	0.003 ± 0.002	0.002 ± 0.001	0.002 ± 0.002	0.005 ± 0.003	0.002 ± 0.001
β_{AMP}	AMPK kinase act.	**0.106 ± 0.046**	**0.090 ± 0.044**	**0.105 ± 0.050**	**0.056 ± 0.050**	**0.037 ± 0.034**	**0.050 ± 0.038**

Supplementary Table S10: **Sobol total sensitivity indices for Model 4.** Bold values indicate influential parameters with a mean index greater than or equal to 0.01.

		**max fraction act. AMPKAR**			**Time to half-max**		
**Parameter**	**Function**	**WT**	**LKB1 KO**	**CaMKK2 KO**	**WT**	**LKB1 KO**	**CaMKK2 KO**
k_{OffAMP}	AMP binding	**2.38e9 ± 2.79e9**	**2.69e9 ± 2.91e9**	**0.111 ± 0.112**	**0.070 ± 0.055**	**0.044 ± 0.033**	**0.139 ± 0.042**
k_{OffADP}	ADP binding	**0.014 ± 0.010**	**0.014 ± 0.011**	**0.025 ± 0.034**	**0.020 ± 0.022**	0.006 ± 0.006	**0.019 ± 0.010**
k_{OffATP}	ATP binding	**0.860 ± 3.147**	**0.852 ± 2.769**	**0.240 ± 0.179**	**0.601 ± 0.980**	**0.604 ± 1.048**	**0.263 ± 0.094**
$K_{m,\text{CaMKK}}$	AMPK phos.	**0.341 ± 0.372**	**0.344 ± 0.343**	0.000 ± 0.000	**0.166 ± 0.176**	**0.210 ± 0.165**	0.000 ± 0.000
k_{LKB1}	AMPK phos.	0.000 ± 0.000	0.000 ± 0.000	**0.324 ± 0.220**	**0.039 ± 0.046**	0.000 ± 0.000	**0.404 ± 0.113**
$K_{\text{m,LKB1}}$	AMPK phos.	0.000 ± 0.000	0.000 ± 0.000	**0.223 ± 0.184**	**0.053 ± 0.060**	0.000 ± 0.000	**0.184 ± 0.060**
k_{PP}	AMPK dephos.	0.000 ± 0.000	0.000 ± 0.000	**0.179 ± 0.141**	**0.046 ± 0.053**	0.000 ± 0.000	**0.177 ± 0.083**
$K_{\text{M,PP}}$	AMPK dephos.	**0.014 ± 0.010**	**0.014 ± 0.009**	**0.074 ± 0.070**	**0.010 ± 0.005**	0.007 ± 0.004	**0.023 ± 0.011**
α_{LKB1}	AMPK phos.	**0.475 ± 0.455**	**0.479 ± 0.412**	**0.107 ± 0.107**	**0.212 ± 0.228**	**0.224 ± 0.254**	**0.051 ± 0.034**
α_{PP}	AMPK dephos.	**0.457 ± 0.431**	**0.461 ± 0.392**	**0.129 ± 0.135**	**0.200 ± 0.206**	**0.204 ± 0.232**	**0.039 ± 0.023**
β_{AMP}	AMPK kinase act.	**0.882 ± 0.471**	**0.887 ± 0.416**	**0.385 ± 0.284**	**0.573 ± 0.261**	**0.618 ± 0.289**	**0.206 ± 0.054**

Supplementary Table S11: **Sobol total sensitivity indices for Model 5.** Bold values indicate influential parameters with a mean index greater than or equal to 0.01.

		**max fraction act. AMPKAR**			**Time to half-max**		
**Parameter**	**Function**	**WT**	**LKB1 KO**	**CaMKK2 KO**	**WT**	**LKB1 KO**	**CaMKK2 KO**
k_{OffAMP}	AMP binding	**0.092 ± 0.027**	**0.089 ± 0.023**	**0.090 ± 0.026**	**0.181 ± 0.102**	**0.136 ± 0.111**	**0.169 ± 0.081**
k_{OffADP}	ADP binding	**0.127 ± 0.042**	**0.054 ± 0.023**	**0.125 ± 0.044**	**0.195 ± 0.146**	**0.082 ± 0.059**	**0.186 ± 0.118**
k_{OffATP}	ATP binding	**0.246 ± 0.059**	**0.131 ± 0.048**	**0.249 ± 0.076**	**0.447 ± 0.269**	**0.148 ± 0.100**	**0.425 ± 0.188**
$k_{OffCaMKK}$	NaN	0.000 ± 0.000	0.002 ± 0.001	0.000 ± 0.000	0.001 ± 0.001	0.005 ± 0.003	0.000 ± 0.000
$k_{PhosCaMKK}$	AMPK phos.	0.002 ± 0.002	**0.011 ± 0.007**	0.000 ± 0.000	0.004 ± 0.002	**0.014 ± 0.009**	0.000 ± 0.000
$k_{OffLKB1}$	AMPK phos.	0.003 ± 0.002	0.000 ± 0.000	0.006 ± 0.003	**0.014 ± 0.007**	0.000 ± 0.000	**0.021 ± 0.010**
$k_{PhosLKB1}$	AMPK phos.	**0.012 ± 0.008**	0.000 ± 0.000	**0.023 ± 0.010**	**0.037 ± 0.012**	0.000 ± 0.000	**0.060 ± 0.025**
k_{OffPP}	AMPK dephos.	**0.036 ± 0.019**	**0.036 ± 0.012**	**0.035 ± 0.018**	**0.457 ± 0.268**	**0.549 ± 0.367**	**0.375 ± 0.219**
$k_{DephosPP}$	AMPK dephos.	**0.026 ± 0.019**	**0.070 ± 0.023**	**0.027 ± 0.020**	**0.084 ± 0.038**	**0.193 ± 0.177**	**0.085 ± 0.049**
$k_{OffAMPK}$	AMPK kinase act.	**0.024 ± 0.013**	**0.031 ± 0.019**	**0.024 ± 0.013**	**0.156 ± 0.081**	**0.183 ± 0.193**	**0.155 ± 0.090**
$k_{PhosAMPK}$	AMPK kinase act.	**0.197 ± 0.058**	**0.118 ± 0.041**	**0.187 ± 0.050**	**0.321 ± 0.115**	**0.061 ± 0.040**	**0.318 ± 0.140**
k_{OffPP1}	AMKPAR dephos.	**0.095 ± 0.025**	**0.084 ± 0.024**	**0.090 ± 0.025**	**0.191 ± 0.096**	**0.050 ± 0.052**	**0.196 ± 0.110**
$k_{DephosPP1}$	AMKPAR dephos.	**0.398 ± 0.094**	**0.500 ± 0.098**	**0.387 ± 0.088**	**0.328 ± 0.144**	**0.066 ± 0.049**	**0.311 ± 0.130**
α_{PP}	AMPK dephos.	**0.040 ± 0.018**	**0.113 ± 0.041**	**0.043 ± 0.019**	**0.187 ± 0.194**	**0.369 ± 0.232**	**0.143 ± 0.136**
β_{AMP}	AMPK kinase act.	**0.071 ± 0.030**	**0.046 ± 0.024**	**0.069 ± 0.033**	**0.086 ± 0.041**	**0.012 ± 0.011**	**0.085 ± 0.043**
β_{LKB1}	AMPK phos.	**0.022 ± 0.011**	0.010 ± 0.009	**0.044 ± 0.027**	**0.028 ± 0.023**	0.004 ± 0.004	**0.038 ± 0.026**
β_{CaMKK}	AMPK phos.	0.000 ± 0.000	0.000 ± 0.000	0.000 ± 0.000	0.000 ± 0.000	0.000 ± 0.000	0.000 ± 0.000

Supplementary Table S12: **Sobol total sensitivity indices for Model 6.** Bold values indicate influential parameters with a mean index greater than or equal to 0.01.

		**max fraction act. AMPKAR**			**Time to half-max**		
**Parameter**	**Function**	**WT**	**LKB1 KO**	**CaMKK2 KO**	**WT**	**LKB1 KO**	**CaMKK2 KO**
k_{OffAMP}	AMP binding	**0.196 ± 0.071**	**0.261 ± 0.075**	**0.198 ± 0.072**	**0.271 ± 0.060**	**0.401 ± 0.171**	**0.270 ± 0.055**
k_{OffADP}	ADP binding	**0.174 ± 0.080**	**0.075 ± 0.035**	**0.173 ± 0.062**	**0.114 ± 0.037**	**0.076 ± 0.050**	**0.114 ± 0.034**
k_{OffATP}	ATP binding	**0.271 ± 0.084**	**0.139 ± 0.059**	**0.273 ± 0.086**	**0.414 ± 0.126**	**0.243 ± 0.160**	**0.412 ± 0.117**
$K_{m,\mathrm{CaMKK}}$	AMPK phos.	0.000 ± 0.000	**0.311 ± 0.110**	0.000 ± 0.000	0.000 ± 0.000	**0.403 ± 0.249**	0.000 ± 0.000
k_{LKB1}	AMPK phos.	**0.018 ± 0.029**	0.000 ± 0.000	**0.020 ± 0.037**	**0.016 ± 0.010**	0.000 ± 0.000	**0.016 ± 0.010**
$K_{\mathrm{m,LKB1}}$	AMPK phos.	**0.017 ± 0.029**	0.000 ± 0.000	**0.020 ± 0.037**	0.006 ± 0.007	0.000 ± 0.000	0.006 ± 0.007
k_{PP}	AMPK dephos.	0.000 ± 0.000	0.003 ± 0.002	0.000 ± 0.001	0.000 ± 0.000	**0.054 ± 0.038**	0.000 ± 0.000
$K_{\mathrm{M,PP}}$	AMPK dephos.	0.004 ± 0.004	0.006 ± 0.003	0.003 ± 0.004	0.001 ± 0.001	**0.233 ± 0.116**	0.001 ± 0.001
α_{PP}	AMPK dephos.	**0.598 ± 0.127**	**0.271 ± 0.108**	**0.595 ± 0.130**	**0.595 ± 0.110**	**0.250 ± 0.161**	**0.595 ± 0.097**
β_{AMP}	AMPK kinase act.	0.001 ± 0.001	0.000 ± 0.000	0.001 ± 0.001	0.002 ± 0.002	0.000 ± 0.000	0.002 ± 0.002
β_{LKB1}	AMPK phos.	0.000 ± 0.000	**0.258 ± 0.095**	0.000 ± 0.000	0.000 ± 0.000	**0.392 ± 0.237**	0.000 ± 0.000
β_{CaMKK}	AMPK phos.	0.000 ± 0.000	0.000 ± 0.000	0.000 ± 0.000	0.000 ± 0.000	**0.012 ± 0.007**	0.000 ± 0.000

Supplementary Table S13: **Flux terms for metabolism model used in all six AMPK models.**

**Name**	**Flux**
Glycolysis	$J_{\mathrm{gly}} = k_{\mathrm{Gly}}[\mathrm{ADP}]$
ATP hydrolysis	$J_{\mathrm{hydro}} = k_{\mathrm{Hydro}}[\mathrm{ATP}]$
Adenylate kinase	$J_{\mathrm{AK}} = \frac{\frac{V_{\mathrm{forAK}}[\mathrm{ATP}][\mathrm{AMP}]}{(kmt)(kmm)} - \frac{V_{\mathrm{revAK}}[\mathrm{ADP}]^2}{kmd^2}}{1+\frac{[\mathrm{ATP}]}{kmt}+\frac{[\mathrm{AMP}]}{kmm}+\frac{[\mathrm{ATP}][\mathrm{AMP}]}{(kmt)(kmm)}+\frac{2[\mathrm{ADP}]}{kmd}+\frac{[\mathrm{ADP}]^2}{kmd^2}}$
Oxidative phosphorylation	$J_{\mathrm{oxphos}} = \frac{V_{\mathrm{maxOxphos}},(\frac{[\mathrm{ADP}]}{K_{[\mathrm{ADP}]}})^n}{1+(\frac{[\mathrm{ADP}]}{K_{[\mathrm{ADP}]}})^n}$
Creatine kinase	$J_{\mathrm{CK}} = \frac{\frac{V_{\mathrm{revCK}}[\mathrm{ATP}](T_{\mathrm{Cr}}-[\mathrm{PCr}])}{K_{\mathrm{iq}}K_{\mathrm{p}}} - \frac{V_{\mathrm{forCK}}[\mathrm{ADP}][\mathrm{PCr}]}{K_{\mathrm{ia}}K_{\mathrm{b}}}}{1+\frac{[\mathrm{ADP}]}{K_{\mathrm{ia}}}+\frac{[\mathrm{PCr}]}{K_{\mathrm{ib}}}+\frac{[\mathrm{ATP}]}{K_{\mathrm{iq}}}+\frac{[\mathrm{ADP}][\mathrm{PCr}]}{K_{\mathrm{ia}}K_{\mathrm{b}}}+\frac{(T_{\mathrm{Cr}}-[\mathrm{PCr}])[\mathrm{ATP}]}{K_{\mathrm{iq}}K_{\mathrm{p}}}}$

Supplementary Table S14: **Flux terms for calcium-induced CaMKK2 activity model used in all AMPK six models.**

**Name**	**Flux**
Calcium-calmodulim	$J_{\mathrm{Ca}} = k_{\mathrm{on,CaM}}[\mathrm{Ca}]^3[\mathrm{CaM}] - k_{\mathrm{off,CaM}}[\mathrm{CaCaM}]$
CaMKK2 phosphorylation	$J_{\mathrm{CaMKK\text{-}act}} = \frac{k_{\mathrm{phos,CaM}}[\mathrm{CaCaM}]^4[\mathrm{CaMKK}]}{k^4_{\mathrm{mCaM}}+[\mathrm{CaCaM}]^4}$
CaMKK2 dephosphorylation	$J_{\mathrm{CaMKK\text{-}dephos}} = k_{\mathrm{dephos,CaMKK}}[\mathrm{CaMKKp}]$

Supplementary Table S15: **Flux terms for model 1.** Superscripts indicate model number.

**Name**	**Flux**
AMP binding	$J_1^1 = k_{\text{OnAMP}}[\text{AMP}][\text{AMPK}] - k_{\text{OffAMP}}[\text{AMP–AMPK}]$
ADP binding	$J_2^1 = k_{\text{OnADP}}[\text{ADP}][\text{AMPK}] - k_{\text{OffADP}}[\text{ADP–AMPK}]$
ATP binding	$J_3^1 = k_{\text{OnATP}}[\text{ATP}][\text{AMPK}] - k_{\text{OffATP}}[\text{ATP–AMPK}]$
AMP binding	$J_4^1 = k_{\text{OnAMP}}[\text{AMP}][\text{pAMPK}] - k_{\text{OffAMP}}[\text{AMP–pAMPK}]$
ADP binding	$J_5^1 = k_{\text{OnADP}}[\text{ADP}][\text{pAMPK}] - k_{\text{OffADP}}[\text{ADP–pAMPK}]$
ATP binding	$J_6^1 = k_{\text{OnATP}}[\text{ATP}][\text{pAMPK}] - k_{\text{OffATP}}[\text{ATP–pAMPK}]$
CaMKK2 binding	$J_7^1 = k_{\text{OnCaMKK}}[\text{CaMKK–act}][\text{AMPK}] - k_{\text{OffCaMKK}}[\text{CaMKK–act–AMPK}]$
CaMKK2 phos.	$J_8^1 = k_{\text{PhosCaMKK}}[\text{CaMKK–act–AMPK}]$
CaMKK2 binding	$J_9^1 = k_{\text{OnCaMKK}}[\text{CaMKK–act}][\text{AMP–AMPK}] - k_{\text{OffCaMKK}}[\text{CaMKK–act–AMP–AMPK}]$
CaMKK2 phos.	$J_{10}^1 = k_{\text{PhosCaMKK}}[\text{CaMKK–act–AMP–AMPK}]$
CaMKK2 binding	$J_{11}^1 = k_{\text{OnCaMKK}}[\text{CaMKK–act}][\text{ADP–AMPK}] - k_{\text{OffCaMKK}}[\text{CaMKK–act–ADP–AMPK}]$
CaMKK2 phos.	$J_{12}^1 = k_{\text{PhosCaMKK}}[\text{CaMKK–act–ADP–AMPK}]$
CaMKK2 binding	$J_{13}^1 = k_{\text{OnCaMKK}}[\text{CaMKK–act}][\text{ATP–AMPK}] - k_{\text{OffCaMKK}}[\text{CaMKK–act–ATP–AMPK}]$
CaMKK2 phos.	$J_{14}^1 = k_{\text{PhosCaMKK}}[\text{CaMKK–act–ATP–AMPK}]$
LKB1 binding	$J_{15}^1 = k_{\text{OnLKB1}}[\text{LKB1}][\text{AMP–AMPK}] - k_{\text{OffLKB1}}[\text{LKB1–AMP–AMPK}]$
LKB1 phos.	$J_{16}^1 = k_{\text{PhosLKB1}}[\text{LKB1–AMP–AMPK}]$
LKB1 binding	$J_{17}^1 = k_{\text{OnLKB1}}[\text{LKB1}][\text{ADP–AMPK}] - k_{\text{OffLKB1}}[\text{LKB1–ADP–AMPK}]$
LKB1 phos.	$J_{18}^1 = k_{\text{PhosLKB1}}[\text{LKB1–ADP–AMPK}]$
PPase binding	$J_{19}^1 = k_{\text{OnPP}}[\text{PP}][\text{pAMPK}] - k_{\text{OffPP}}[\text{PP–pAMPK}]$
PPase dephos.	$J_{20}^1 = k_{\text{DephosPP}}[\text{PP–pAMPK}]$
PPase binding	$J_{21}^1 = k_{\text{OnPP}}[\text{PP}][\text{ATP–pAMPK}] - k_{\text{OffPP}}[\text{PP–ATP–pAMPK}]$
PPase dephos.	$J_{22}^1 = k_{\text{DephosPP}}[\text{PP–ATP–pAMPK}]$
AMPK binding	$J_{23}^1 = k_{\text{OnAMPK}}[\text{AMPKAR}][\text{AMP–pAMPK}] - k_{\text{OffAMPK}}[\text{AMPKAR–AMP–pAMPK}]$
AMPK phos.	$J_{24}^1 = k_{\text{PhosAMPK}}[\text{AMPKAR–AMP–pAMPK}]$
PPase binding	$J_{25}^1 = k_{\text{OnPP1}}[\text{PP1}][\text{pAMPKAR}] - k_{\text{OffPP1}}[\text{PP1–pAMPKAR}]$
PPase dephos.	$J_{26}^1 = k_{\text{DephosPP1}}[\text{PP1–pAMPKAR}]$
AMP binding	$J_{27}^1 = k_{\text{OnAMP}}[\text{AMP}][\text{CaMKK–act–AMPK}] - k_{\text{OffADP}}[\text{CaMKK–act–AMP–AMPK}]$
ADP binding	$J_{28}^1 = k_{\text{OnADP}}[\text{ADP}][\text{CaMKK–act–AMPK}] - k_{\text{OffADP}}[\text{CaMKK–act–ADP–AMPK}]$
ATP binding	$J_{29}^1 = k_{\text{OnATP}}[\text{ATP}][\text{CaMKK–act–AMPK}] - k_{\text{OffATP}}[\text{CaMKK–act–ATP–AMPK}]$
ATP binding	$J_{30}^1 = k_{\text{OnATP}}[\text{ATP}][\text{PP–pAMPK}] - k_{\text{OffATP}}[\text{PP–ATP–pAMPK}]$

Supplementary Table S16: **Flux terms for model 2.** Superscripts indicate model number.

**Name**	**Flux**
AMP binding	$J_1^2 = k_{\mathrm{OnAMP}}[\mathrm{AMP}][\mathrm{AMPK}] - k_{\mathrm{OffAMP}}[\mathrm{AMP–AMPK}]$
ADP binding	$J_2^2 = k_{\mathrm{OnADP}}[\mathrm{ADP}][\mathrm{AMPK}] - k_{\mathrm{OffADP}}[\mathrm{ADP–AMPK}]$
ATP binding	$J_3^2 = k_{\mathrm{OnATP}}[\mathrm{ATP}][\mathrm{AMPK}] - k_{\mathrm{OffATP}}[\mathrm{ATP–AMPK}]$
AMP binding	$J_4^2 = k_{\mathrm{OnAMP}}[\mathrm{AMP}][\mathrm{pAMPK}] - k_{\mathrm{OffAMP}}[\mathrm{AMP–pAMPK}]$
ADP binding	$J_5^2 = k_{\mathrm{OnADP}}[\mathrm{ADP}][\mathrm{pAMPK}] - k_{\mathrm{OffADP}}[\mathrm{ADP–pAMPK}]$
ATP binding	$J_6^2 = k_{\mathrm{OnATP}}[\mathrm{ATP}][\mathrm{pAMPK}] - k_{\mathrm{OffATP}}[\mathrm{ATP–pAMPK}]$
CaMKK2 phos.	$J_7^2 = \frac{k_{\mathrm{CaMKK}}[\mathrm{CaMKK–act}][\mathrm{AMPK}]}{K_{\mathrm{m,CaMKK}}+[\mathrm{AMPK}]}$
CaMKK2 phos.	$J_8^2 = \frac{k_{\mathrm{CaMKK}}[\mathrm{CaMKK–act}][\mathrm{AMP–AMPK}]}{K_{\mathrm{m,CaMKK}}+[\mathrm{AMP–AMPK}]}$
CaMKK2 phos.	$J_9^2 = \frac{k_{\mathrm{CaMKK}}[\mathrm{CaMKK–act}][\mathrm{ADP–AMPK}]}{K_{\mathrm{m,CaMKK}}+[\mathrm{ADP–AMPK}]}$
CaMKK2 phos.	$J_{10}^2 = \frac{k_{\mathrm{CaMKK}}[\mathrm{CaMKK–act}][\mathrm{ATP–AMPK}]}{K_{\mathrm{m,CaMKK}}+[\mathrm{ATP–AMPK}]}$
LKB1 phos.	$J_{11}^2 = \frac{k_{\mathrm{LKB1}}\mathrm{LKB1}_{\mathrm{tot}}[\mathrm{AMP–AMPK}]}{K_{\mathrm{m,LKB1}}+[\mathrm{AMP–AMPK}]}$
LKB1 phos.	$J_{12}^2 = \frac{k_{\mathrm{LKB1}}\mathrm{LKB1}_{\mathrm{tot}}[\mathrm{ADP–AMPK}]}{K_{\mathrm{m,LKB1}}+[\mathrm{ADP–AMPK}]}$
PPase dephos.	$J_{13}^2 = \frac{k_{\mathrm{PP}}\mathrm{PP}_{\mathrm{tot}}[\mathrm{pAMPK}]}{K_{\mathrm{m,PP}}+[\mathrm{pAMPK}]}$
PPase dephos.	$J_{14}^2 = \frac{k_{\mathrm{PP}}\mathrm{PP}_{\mathrm{tot}}[\mathrm{ATP–pAMPK}]}{K_{\mathrm{m,PP}}+[\mathrm{ATP–pAMPK}]}$
AMPK phos.	$J_{15}^2 = \frac{k_{\mathrm{AMPK}}[\mathrm{AMP–pAMPK}][\mathrm{AMPKAR}]}{K_{\mathrm{m,AMPK}}+[\mathrm{AMPKAR}]}$
PPase dephos.	$J_{16}^2 = \frac{k_{\mathrm{PP1}}\mathrm{PP1}_{\mathrm{tot}}[\mathrm{pAMPKAR}]}{K_{\mathrm{m,PP1}}+[\mathrm{pAMPKAR}]}$

Supplementary Table S17: **Flux terms for model 3.** Superscripts indicate model number.

**Name**	**Flux**
AMP binding	$J^3_1 = k_{\text{OnAMP}}[\text{AMP}][\text{AMPK}] - k_{\text{OffAMP}}[\text{AMP–AMPK}]$
ADP binding	$J^3_2 = k_{\text{OnADP}}[\text{ADP}][\text{AMPK}] - k_{\text{OffADP}}[\text{ADP–AMPK}]$
ATP binding	$J^3_3 = k_{\text{OnATP}}[\text{ATP}][\text{AMPK}] - k_{\text{OffATP}}[\text{ATP–AMPK}]$
AMP binding	$J^3_4 = k_{\text{OnAMP}}[\text{AMP}][\text{pAMPK}] - k_{\text{OffAMP}}[\text{AMP–pAMPK}]$
ADP binding	$J^3_5 = k_{\text{OnADP}}[\text{ADP}][\text{pAMPK}] - k_{\text{OffADP}}[\text{ADP–pAMPK}]$
ATP binding	$J^3_6 = k_{\text{OnATP}}[\text{ATP}][\text{pAMPK}] - k_{\text{OffATP}}[\text{ATP–pAMPK}]$
CaMKK2 binding	$J^3_7 = k_{\text{OnCaMKK}}[\text{CaMKK–act}][\text{AMPK}] - k_{\text{OffCaMKK}}[\text{CaMKK–act–AMPK}]$
CaMKK2 phos.	$J^3_8 = k_{\text{PhosCaMKK}}[\text{CaMKK–act–AMPK}]$
CaMKK2 binding	$J^3_9 = k_{\text{OnCaMKK}}[\text{CaMKK–act}][\text{AMP–AMPK}] - k_{\text{OffCaMKK}}[\text{CaMKK–act–AMP–AMPK}]$
AMP binding	$J^3_{10} = k_{\text{OnAMP}}[\text{AMP}][\text{CaMKK–act–AMPK}] - k_{\text{OffAMP}}[\text{CaMKK–act–AMP–AMPK}]$
CaMKK2 phos.	$J^3_{11} = k_{\text{PhosCaMKK}}[\text{CaMKK–act–AMP–AMPK}]$
CaMKK2 binding	$J^3_{12} = k_{\text{OnCaMKK}}[\text{CaMKK–act}][\text{ADP–AMPK}] - k_{\text{OffCaMKK}}[\text{CaMKK–act–ADP–AMPK}]$
ADP binding	$J^3_{13} = k_{\text{OnADP}}[\text{ADP}][\text{CaMKK–act–AMPK}] - k_{\text{OffADP}}[\text{CaMKK–act–ADP–AMPK}]$
CaMKK2 phos.	$J^3_{14} = k_{\text{PhosCaMKK}}[\text{CaMKK–act–ADP–AMPK}]$
CaMKK2 binding	$J^3_{15} = k_{\text{OnCaMKK}}[\text{CaMKK–act}][\text{ATP–AMPK}] - k_{\text{OffCaMKK}}[\text{CaMKK–act–ATP–AMPK}]$
ATP binding	$J^3_{16} = k_{\text{OnATP}}[\text{ATP}][\text{CaMKK–act–AMPK}] - k_{\text{OffATP}}[\text{CaMKK–act–ATP–AMPK}]$
CaMKK2 phos.	$J^3_{17} = k_{\text{PhosCaMKK}}[\text{CaMKK–act–ATP–AMPK}]$
LKB1 binding	$J^3_{18} = k_{\text{OnLKB1}}[\text{LKB1}][\text{AMPK}] - k_{\text{OffLKB1}}[\text{LKB1–AMPK}]$
LKB1 phos.	$J^3_{19} = k_{\text{PhosLKB1}}[\text{LKB1–AMPK}]$
LKB1 binding	$J^3_{20} = k_{\text{OnLKB1}}[\text{LKB1}][\text{AMP–AMPK}] - \alpha_{\text{LKB1}}k_{\text{OffLKB1}}[\text{LKB1–AMP–AMPK}]$
AMP binding	$J^3_{21} = k_{\text{OnAMP}}[\text{AMP}][\text{LKB1–AMPK}] - k_{\text{OffAMP}}[\text{LKB1–AMP–AMPK}]$
LKB1 phos.	$J^3_{22} = k_{\text{PhosLKB1}}[\text{LKB1–AMP–AMPK}]$
LKB1 binding	$J^3_{23} = k_{\text{OnLKB1}}[\text{LKB1}][\text{ADP–AMPK}] - \alpha_{\text{LKB1}}k_{\text{OffLKB1}}[\text{LKB1–ADP–AMPK}]$
ADP binding	$J^3_{24} = k_{\text{OnADP}}[\text{ADP}][\text{LKB1–AMPK}] - k_{\text{OffADP}}[\text{LKB1–ADP–AMPK}]$
LKB1 phos.	$J^3_{25} = k_{\text{PhosLKB1}}[\text{LKB1–ADP–AMPK}]$
PPase binding	$J^3_{26} = k_{\text{OnPP}}[\text{PP}][\text{pAMPK}] - k_{\text{OffPP}}[\text{PP–pAMPK}]$
PPase dephos.	$J^3_{27} = k_{\text{DephosPP}}[\text{PP–pAMPK}]$
PPase binding	$J^3_{28} = k_{\text{OnPP}}[\text{PP}][\text{AMP–pAMPK}] - \alpha_{\text{PP}}k_{\text{OffPP}}[\text{PP–AMP–pAMPK}]$
AMP binding	$J^3_{29} = k_{\text{OnAMP}}[\text{AMP}][\text{PP–pAMPK}] - k_{\text{OffAMP}}[\text{PP–AMP–pAMPK}]$
PPase dephos.	$J^3_{30} = k_{\text{DephosPP}}[\text{PP–AMP–pAMPK}]$
PPase binding	$J^3_{31} = k_{\text{OnPP}}[\text{PP}][\text{ADP–pAMPK}] - \alpha_{\text{PP}}k_{\text{OffPP}}[\text{PP–ADP–pAMPK}]$
ADP binding	$J^3_{32} = k_{\text{OnADP}}[\text{ADP}][\text{PP–pAMPK}] - k_{\text{OffADP}}[\text{PP–ADP–pAMPK}]$
PPase dephos.	$J^3_{33} = k_{\text{DephosPP}}[\text{PP–ADP–pAMPK}]$
PPase binding	$J^3_{34} = k_{\text{OnPP}}[\text{PP}][\text{ATP–pAMPK}] - k_{\text{OffPP}}[\text{PP–ATP–pAMPK}]$
ATP binding	$J^3_{35} = k_{\text{OnATP}}[\text{ATP}][\text{PP–pAMPK}] - k_{\text{OffATP}}[\text{PP–ATP–pAMPK}]$
PPase dephos.	$J^3_{36} = k_{\text{DephosPP}}[\text{PP–ATP–pAMPK}]$
AMPK binding	$J^3_{37} = k_{\text{OnAMPK}}[\text{AMPKAR}][\text{pAMPK}] - k_{\text{OffAMPK}}[\text{AMPKAR–pAMPK}]$
AMPK phos.	$J^3_{38} = k_{\text{PhosAMPK}}[\text{AMPKAR–pAMPK}]$
AMPK binding	$J^3_{39} = k_{\text{OnAMPK}}[\text{AMPKAR}][\text{AMP–pAMPK}] - k_{\text{OffAMPK}}[\text{AMPKAR–AMP–pAMPK}]$
AMP binding	$J^3_{40} = k_{\text{OnAMP}}[\text{AMP}][\text{AMPKAR–pAMPK}] - k_{\text{OffAMP}}[\text{AMPKAR–AMP–pAMPK}]$
AMP binding	$J^3_{41} = \beta_{\text{AMP}}k_{\text{PhosAMPK}}[\text{AMPKAR–AMP–pAMPK}]$
AMPK binding	$J^3_{42} = k_{\text{OnAMPK}}[\text{AMPKAR}][\text{ADP–pAMPK}] - k_{\text{OffAMPK}}[\text{AMPKAR–ADP–pAMPK}]$
ADP binding	$J^3_{43} = k_{\text{OnADP}}[\text{ADP}][\text{AMPKAR–pAMPK}] - k_{\text{OffADP}}[\text{AMPKAR–ADP–pAMPK}]$
AMPK phos.	$J^3_{44} = k_{\text{PhosAMPK}}[\text{AMPKAR–ADP–pAMPK}]$
PPase binding	$J^3_{45} = k_{\text{OnPP1}}[\text{pAMPKAR}][\text{PP1}] - k_{\text{OffPP1}}[\text{PP1–pAMPKAR}]$
PPase dephos.	$J^3_{46} = k_{\text{DephosPP1}}[\text{PP1–pAMPKAR}]$

Supplementary Table S18: **Flux terms for model 4.** Superscripts indicate model number.

**Name**	**Flux**
AMP binding	$J_1^4 = k_{\text{OnAMP}}[\text{AMP}][\text{AMPK}] - k_{\text{OffAMP}}[\text{AMP–AMPK}]$
ADP binding	$J_2^4 = k_{\text{OnADP}}[\text{ADP}][\text{AMPK}] - k_{\text{OffADP}}[\text{ADP–AMPK}]$
ATP binding	$J_3^4 = k_{\text{OnATP}}[\text{ATP}][\text{AMPK}] - k_{\text{OffATP}}[\text{ATP–AMPK}]$
AMP binding	$J_4^4 = k_{\text{OnAMP}}[\text{AMP}][\text{pAMPK}] - k_{\text{OffAMP}}[\text{AMP–pAMPK}]$
ADP binding	$J_5^4 = k_{\text{OnADP}}[\text{ADP}][\text{pAMPK}] - k_{\text{OffADP}}[\text{ADP–pAMPK}]$
ATP binding	$J_6^4 = k_{\text{OnATP}}[\text{ATP}][\text{pAMPK}] - k_{\text{OffATP}}[\text{ATP–pAMPK}]$
CaMKK2 phos.	$J_7^4 = \frac{k_{\text{CaMKK}}[\text{CaMKK–act}][\text{AMPK}]}{K_{\text{m,CaMKK}}+[\text{AMPK}]}$
CaMKK2 phos.	$J_8^4 = \frac{k_{\text{CaMKK}}[\text{CaMKK–act}][\text{AMP–AMPK}]}{K_{\text{m,CaMKK}}+[\text{AMP–AMPK}]}$
CaMKK2 phos.	$J_9^4 = \frac{k_{\text{CaMKK}}[\text{CaMKK–act}][\text{ADP–AMPK}]}{K_{\text{m,CaMKK}}+[\text{ADP–AMPK}]}$
CaMKK2 phos.	$J_{10}^4 = \frac{k_{\text{CaMKK}}[\text{CaMKK–act}][\text{ATP–AMPK}]}{K_{\text{m,CaMKK}}+[\text{ATP–AMPK}]}$
LKB1 phos.	$J_{11}^4 = \frac{k_{\text{LKB1}}\text{LKB1}_{\text{tot}}[\text{AMPK}]}{K_{\text{m,LKB1}}+[\text{AMPK}]}$
LKB1 phos.	$J_{12}^4 = \frac{k_{\text{LKB1}}\text{LKB1}_{\text{tot}}[\text{AMP–AMPK}]}{\alpha_{\text{LKB1}}K_{\text{m,LKB1}}+[\text{AMP–AMPK}]}$
LKB1 phos.	$J_{13}^4 = \frac{k_{\text{LKB1}}\text{LKB1}_{\text{tot}}[\text{ADP–AMPK}]}{\alpha_{\text{LKB1}}K_{\text{m,LKB1}}+[\text{ADP–AMPK}]}$
PPase dephos.	$J_{14}^4 = \frac{k_{\text{PP}}\text{PP}_{\text{tot}}[\text{pAMPK}]}{K_{\text{m,PP}}+[\text{pAMPK}]}$
PPase dephos.	$J_{15}^4 = \frac{k_{\text{PP}}\text{PP}_{\text{tot}}[\text{AMP–pAMPK}]}{\alpha_{\text{PP}}K_{\text{m,PP}}+[\text{AMP–pAMPK}]}$
PPase dephos.	$J_{16}^4 = \frac{k_{\text{PP}}\text{PP}_{\text{tot}}[\text{ADP–pAMPK}]}{\alpha_{\text{PP}}K_{\text{m,PP}}+[\text{ADP–pAMPK}]}$
PPase dephos.	$J_{17}^4 = \frac{k_{\text{PP}}\text{PP}_{\text{tot}}[\text{ATP–pAMPK}]}{K_{\text{m,PP}}+[\text{ATP–pAMPK}]}$
AMPK phos.	$J_{18}^4 = \frac{k_{\text{AMPK}}[\text{pAMPK}][\text{AMPKAR}]}{K_{\text{m,AMPK}}+[\text{AMPKAR}]}$
AMPK phos.	$J_{19}^4 = \frac{\beta_{\text{AMP}}k_{\text{AMPK}}[\text{AMP–pAMPK}][\text{AMPKAR}]}{K_{\text{m,AMPK}}+[\text{AMPKAR}]}$
AMPK phos.	$J_{20}^4 = \frac{k_{\text{AMPK}}[\text{ADP–pAMPK}][\text{AMPKAR}]}{K_{\text{m,AMPK}}+[\text{AMPKAR}]}$
PPase dephos.	$J_{21}^4 = \frac{k_{\text{PP1}}\text{PP1}_{\text{tot}}[\text{pAMPKAR}]}{K_{\text{m,PP1}}+[\text{pAMPKAR}]}$

Supplementary Table S19: **Flux terms for model 5.** Superscripts indicate model number.

Name	Flux
AMP binding	$J_1^5 = k_{\mathrm{OnAMP}}[\mathrm{AMP}][\mathrm{AMPK}] - k_{\mathrm{OffAMP}}[\mathrm{AMP–AMPK}]$
ADP binding	$J_2^5 = k_{\mathrm{OnADP}}[\mathrm{ADP}][\mathrm{AMPK}] - k_{\mathrm{OffADP}}[\mathrm{ADP–AMPK}]$
ATP binding	$J_3^5 = k_{\mathrm{OnATP}}[\mathrm{ATP}][\mathrm{AMPK}] - k_{\mathrm{OffATP}}[\mathrm{ATP–AMPK}]$
AMP binding	$J_4^5 = k_{\mathrm{OnAMP}}[\mathrm{AMP}][\mathrm{pAMPK}] - k_{\mathrm{OffAMP}}[\mathrm{AMP–pAMPK}]$
ADP binding	$J_5^5 = k_{\mathrm{OnADP}}[\mathrm{ADP}][\mathrm{pAMPK}] - k_{\mathrm{OffADP}}[\mathrm{ADP–pAMPK}]$
ATP binding	$J_6^5 = k_{\mathrm{OnATP}}[\mathrm{ATP}][\mathrm{pAMPK}] - k_{\mathrm{OffATP}}[\mathrm{ATP–pAMPK}]$
CaMKK2 binding	$J_7^5 = k_{\mathrm{OnCaMKK}}[\mathrm{CaMKK–act}][\mathrm{AMPK}] - k_{\mathrm{OffCaMKK}}[\mathrm{CaMKK–act–AMPK}]$
CaMKK2 phos.	$J_8^5 = k_{\mathrm{PhosCaMKK}}[\mathrm{CaMKK–act–AMPK}]$
CaMKK2 binding	$J_9^5 = k_{\mathrm{OnCaMKK}}[\mathrm{CaMKK–act}][\mathrm{AMP–AMPK}] - k_{\mathrm{OffCaMKK}}[\mathrm{CaMKK–act–AMP–AMPK}]$
CaMKK2 phos.	$J_{10}^5 = \beta_{\mathrm{CaMKK}} k_{\mathrm{PhosCaMKK}}[\mathrm{CaMKK–act–AMP–AMPK}]$
CaMKK2 binding	$J_{11}^5 = k_{\mathrm{OnCaMKK}}[\mathrm{CaMKK–act}][\mathrm{ADP–AMPK}] - k_{\mathrm{OffCaMKK}}[\mathrm{CaMKK–act–ADP–AMPK}]$
CaMKK2 phos.	$J_{12}^5 = \beta_{\mathrm{CaMKK}} k_{\mathrm{PhosCaMKK}}[\mathrm{CaMKK–act–ADP–AMPK}]$
CaMKK2 binding	$J_{13}^5 = k_{\mathrm{OnCaMKK}}[\mathrm{CaMKK–act}][\mathrm{ATP–AMPK}] - k_{\mathrm{OffCaMKK}}[\mathrm{CaMKK–act–ATP–AMPK}]$
CaMKK2 phos.	$J_{14}^5 = k_{\mathrm{PhosCaMKK}}[\mathrm{CaMKK–act–ATP–AMPK}]$
LKB1 binding	$J_{15}^5 = k_{\mathrm{OnLKB1}}[\mathrm{LKB1}][\mathrm{AMP–AMPK}] - k_{\mathrm{OffLKB1}}[\mathrm{LKB1–AMP–AMPK}]$
LKB1 phos.	$J_{16}^5 = \beta_{\mathrm{LKB1}} k_{\mathrm{PhosLKB1}}[\mathrm{LKB1–AMP–AMPK}]$
LKB1 binding	$J_{17}^5 = k_{\mathrm{OnLKB1}}[\mathrm{LKB1}][\mathrm{ADP–AMPK}] - k_{\mathrm{OffLKB1}}[\mathrm{LKB1–ADP–AMPK}]$
LKB1 phos.	$J_{18}^5 = \beta_{\mathrm{LKB1}} k_{\mathrm{PhosLKB1}}[\mathrm{LKB1–ADP–AMPK}]$
LKB1 binding	$J_{19}^5 = k_{\mathrm{OnLKB1}}[\mathrm{LKB1}][\mathrm{ATP–AMPK}] - k_{\mathrm{OffLKB1}}[\mathrm{LKB1–ATP–AMPK}]$
LKB1 phos.	$J_{20}^5 = k_{\mathrm{PhosLKB1}}[\mathrm{LKB1–ATP–AMPK}]$
LKB1 binding	$J_{21}^5 = k_{\mathrm{OnLKB1}}[\mathrm{LKB1}][\mathrm{AMPK}] - k_{\mathrm{OffLKB1}}[\mathrm{LKB1–AMPK}]$
LKB1 phos.	$J_{22}^5 = k_{\mathrm{PhosLKB1}}[\mathrm{LKB1–AMPK}]$
PPase binding	$J_{23}^5 = k_{\mathrm{OnPP}}[\mathrm{PP}][\mathrm{pAMPK}] - k_{\mathrm{OffPP}}[\mathrm{PP–pAMPK}]$
PPase dephos.	$J_{24}^5 = k_{\mathrm{DephosPP}}[\mathrm{PP–pAMPK}]$
PPase binding	$J_{25}^5 = k_{\mathrm{OnPP}}[\mathrm{PP}][\mathrm{ATP–pAMPK}] - k_{\mathrm{OffPP}}[\mathrm{PP–ATP–pAMPK}]$
PPase dephos.	$J_{26}^5 = k_{\mathrm{DephosPP}}[\mathrm{PP–ATP–pAMPK}]$
PPase binding	$J_{27}^5 = k_{\mathrm{OnPP}}[\mathrm{PP}][\mathrm{AMP–pAMPK}] - k_{\mathrm{OffPP}}[\mathrm{PP–AMP–pAMPK}]$
PPase dephos.	$J_{28}^5 = \alpha_{\mathrm{PP}} k_{\mathrm{DephosPP}}[\mathrm{PP–AMP–pAMPK}]$
PPase binding	$J_{29}^5 = k_{\mathrm{OnPP}}[\mathrm{PP}][\mathrm{ADP–pAMPK}] - k_{\mathrm{OffPP}}[\mathrm{PP–ADP–pAMPK}]$
PPase dephos.	$J_{30}^5 = \alpha_{\mathrm{PP}} k_{\mathrm{DephosPP}}[\mathrm{PP–ADP–pAMPK}]$
AMPK binding	$J_{31}^5 = k_{\mathrm{OnAMPK}}[\mathrm{AMPKAR}][\mathrm{AMP–pAMPK}] - k_{\mathrm{OffAMPK}}[\mathrm{AMPKAR–AMP–pAMPK}]$
AMPK phos.	$J_{32}^5 = \beta_{\mathrm{AMP}} k_{\mathrm{PhosAMPK}}[\mathrm{AMPKAR–AMP–pAMPK}]$
AMPK binding	$J_{33}^5 = k_{\mathrm{OnAMPK}}[\mathrm{AMPKAR}][\mathrm{ADP–pAMPK}] - k_{\mathrm{OffAMPK}}[\mathrm{AMPKAR–ADP–pAMPK}]$
AMPK phos.	$J_{34}^5 = k_{\mathrm{PhosAMPK}}[\mathrm{AMPKAR–ADP–pAMPK}]$
AMPK binding	$J_{35}^5 = k_{\mathrm{OnAMPK}}[\mathrm{AMPKAR}][\mathrm{ATP–pAMPK}] - k_{\mathrm{OffAMPK}}[\mathrm{AMPKAR–ATP–pAMPK}]$
AMPK phos.	$J_{36}^5 = k_{\mathrm{PhosAMPK}}[\mathrm{AMPKAR–ATP–pAMPK}]$
AMPK binding	$J_{37}^5 = k_{\mathrm{OnAMPK}}[\mathrm{AMPKAR}][\mathrm{pAMPK}] - k_{\mathrm{OffAMPK}}[\mathrm{AMPKAR–pAMPK}]$
AMPK phos.	$J_{38}^5 = k_{\mathrm{PhosAMPK}}[\mathrm{AMPKAR–pAMPK}]$
PPase binding	$J_{39}^5 = k_{\mathrm{OnPP1}}[\mathrm{PP1}][\mathrm{pAMPKAR}] - k_{\mathrm{OffPP1}}[\mathrm{PP1–pAMPKAR}]$
PPase dephos.	$J_{40}^5 = k_{\mathrm{DephosPP1}}[\mathrm{PP1–pAMPKAR}]$
AMP binding	$J_{41}^5 = k_{\mathrm{OnAMP}}[\mathrm{AMP}][\mathrm{CaMKK–act–AMPK}] - k_{\mathrm{OffADP}}[\mathrm{CaMKK–act–AMP–AMPK}]$
ADP binding	$J_{42}^5 = k_{\mathrm{OnADP}}[\mathrm{ADP}][\mathrm{CaMKK–act–AMPK}] - k_{\mathrm{OffADP}}[\mathrm{CaMKK–act–ADP–AMPK}]$
ATP binding	$J_{43}^5 = k_{\mathrm{OnATP}}[\mathrm{ATP}][\mathrm{CaMKK–act–AMPK}] - k_{\mathrm{OffATP}}[\mathrm{CaMKK–act–ATP–AMPK}]$
AMP binding	$J_{44}^5 = k_{\mathrm{OnAMP}}[\mathrm{AMP}][\mathrm{LKB1–AMPK}] - k_{\mathrm{OffAMP}}[\mathrm{LKB1–AMP–AMPK}]$
ADP binding	$J_{45}^5 = k_{\mathrm{OnADP}}[\mathrm{ADP}][\mathrm{LKB1–AMPK}] - k_{\mathrm{OffADP}}[\mathrm{LKB1–ADP–AMPK}]$
ATP binding	$J_{46}^5 = k_{\mathrm{OnATP}}[\mathrm{ATP}][\mathrm{LKB1–AMPK}] - k_{\mathrm{OffATP}}[\mathrm{LKB1–ATP–AMPK}]$
AMP binding	$J_{47}^5 = k_{\mathrm{OnAMP}}[\mathrm{AMP}][\mathrm{PP–pAMPK}] - k_{\mathrm{OffAMP}}[\mathrm{PP–AMP–pAMPK}]$
ADP binding	$J_{48}^5 = k_{\mathrm{OnADP}}[\mathrm{ADP}][\mathrm{PP–pAMPK}] - k_{\mathrm{OffADP}}[\mathrm{PP–ADP–pAMPK}]$
ATP binding	$J_{49}^5 = k_{\mathrm{OnATP}}[\mathrm{ATP}][\mathrm{PP–pAMPK}] - k_{\mathrm{OffATP}}[\mathrm{PP–ATP–pAMPK}]$
AMP binding	$J_{50}^5 = k_{\mathrm{OnAMP}}[\mathrm{AMP}][\mathrm{AMPKAR–pAMPK}] - k_{\mathrm{OffAMP}}[\mathrm{AMPKAR–AMP–pAMPK}]$
ADP binding	$J_{51}^5 = k_{\mathrm{OnADP}}[\mathrm{ADP}][\mathrm{AMPKAR–pAMPK}] - k_{\mathrm{OffADP}}[\mathrm{AMPKAR–ADP–pAMPK}]$
ATP binding	$J_{52}^5 = k_{\mathrm{OnATP}}[\mathrm{ATP}][\mathrm{AMPKAR–pAMPK}] - k_{\mathrm{OffATP}}[\mathrm{AMPKAR–ATP–pAMPK}]$

Supplementary Table S20: **Flux terms for model 6.** Superscripts indicate model number.

**Name**	**Flux**
AMP binding	$J_1^6 = k_{\text{OnAMP}}[\text{AMP}][\text{AMPK}] - k_{\text{OffAMP}}[\text{AMP–AMPK}]$
ADP binding	$J_2^6 = k_{\text{OnADP}}[\text{ADP}][\text{AMPK}] - k_{\text{OffADP}}[\text{ADP–AMPK}]$
ATP binding	$J_3^6 = k_{\text{OnATP}}[\text{ATP}][\text{AMPK}] - k_{\text{OffATP}}[\text{ATP–AMPK}]$
AMP binding	$J_4^6 = k_{\text{OnAMP}}[\text{AMP}][\text{pAMPK}] - k_{\text{OffAMP}}[\text{AMP–pAMPK}]$
ADP binding	$J_5^6 = k_{\text{OnADP}}[\text{ADP}][\text{pAMPK}] - k_{\text{OffADP}}[\text{ADP–pAMPK}]$
ATP binding	$J_6^6 = k_{\text{OnATP}}[\text{ATP}][\text{pAMPK}] - k_{\text{OffATP}}[\text{ATP–pAMPK}]$
CaMKK2 phos.	$J_7^6 = \frac{k_{\text{CaMKK}}[\text{CaMKK–act}][\text{AMPK}]}{K_{\text{m,CaMKK}}+[\text{AMPK}]}$
CaMKK2 phos.	$J_8^6 = \frac{\beta_{\text{CaMKK}} k_{\text{CaMKK}}[\text{CaMKK–act}][\text{AMP–AMPK}]}{K_{\text{m,CaMKK}}+[\text{AMP–AMPK}]}$
CaMKK2 phos.	$J_9^6 = \frac{\beta_{\text{CaMKK}} k_{\text{CaMKK}}[\text{CaMKK–act}][\text{ADP–AMPK}]}{K_{\text{m,CaMKK}}+[\text{ADP–AMPK}]}$
CaMKK2 phos.	$J_{10}^6 = \frac{k_{\text{CaMKK}}[\text{CaMKK–act}][\text{ATP–AMPK}]}{K_{\text{m,CaMKK}}+[\text{ATP–AMPK}]}$
LKB1 phos.	$J_{11}^6 = \frac{k_{\text{LKB1}}\text{LKB1}_{\text{tot}}[\text{AMPK}]}{K_{\text{m,LKB1}}+[\text{AMPK}]}$
LKB1 phos.	$J_{12}^6 = \frac{\beta_{\text{LKB1}} k_{\text{LKB1}}\text{LKB1}_{\text{tot}}[\text{AMP–AMPK}]}{K_{\text{m,LKB1}}+[\text{AMP–AMPK}]}$
LKB1 phos.	$J_{13}^6 = \frac{\beta_{\text{LKB1}} k_{\text{LKB1}}\text{LKB1}_{\text{tot}}[\text{ADP–AMPK}]}{K_{\text{m,LKB1}}+[\text{ADP–AMPK}]}$
LKB1 phos.	$J_{14}^6 = \frac{k_{\text{LKB1}}\text{LKB1}_{\text{tot}}[\text{ATP–AMPK}]}{K_{\text{m,LKB1}}+[\text{ATP–AMPK}]}$
PPase dephos	$J_{15}^6 = \frac{k_{\text{PP}}\text{PP}_{\text{tot}}[\text{pAMPK}]}{K_{\text{m,PP}}+[\text{pAMPK}]}$
PPase dephos	$J_{16}^6 = \frac{\alpha_{\text{PP}} k_{\text{PP}}\text{PP}_{\text{tot}}[\text{AMP–pAMPK}]}{K_{\text{m,PP}}+[\text{AMP–pAMPK}]}$
PPase dephos	$J_{17}^6 = \frac{\alpha_{\text{PP}} k_{\text{PP}}\text{PP}_{\text{tot}}[\text{ADP–pAMPK}]}{K_{\text{m,PP}}+[\text{ADP–pAMPK}]}$
PPase dephos	$J_{18}^6 = \frac{k_{\text{PP}}\text{PP}_{\text{tot}}[\text{ATP–pAMPK}]}{K_{\text{m,PP}}+[\text{ATP–pAMPK}]}$
AMPK phos.	$J_{19}^6 = \frac{k_{\text{AMPK}}[\text{pAMPK}][\text{AMPKAR}]}{K_{\text{m,AMPK}}+[\text{AMPKAR}]}$
AMPK phos.	$J_{20}^6 = \frac{\beta_{\text{AMPK}} k_{\text{AMPK}}[\text{AMP–pAMPK}][\text{AMPKAR}]}{K_{\text{m,AMPK}}+[\text{AMPKAR}]}$
AMPK phos.	$J_{21}^6 = \frac{k_{\text{AMPK}}[\text{ADP–pAMPK}][\text{AMPKAR}]}{K_{\text{m,AMPK}}+[\text{AMPKAR}]}$
AMPK phos.	$J_{22}^6 = \frac{k_{\text{AMPK}}[\text{ATP–pAMPK}][\text{AMPKAR}]}{K_{\text{m,AMPK}}+[\text{AMPKAR}]}$
PPase dephos	$J_{23}^6 = \frac{k_{\text{PP1}}\text{PP1}_{\text{tot}}[\text{pAMPKAR}]}{K_{\text{m,PP1}}+[\text{pAMPKAR}]}$

Supplementary Table S21: **Equations for model 1.**

Name	ODE
[AMP]	$-J_1^1 - J_4^1 - J_{\mathrm{AK}} - J_{27}^1$
[ADP]	$-J_2^1 - J_5^1 - J_{\mathrm{gly}} + 2J_{\mathrm{AK}} + J_{\mathrm{hydro}} - J_{\mathrm{oxphos}} + J_{\mathrm{CK}} - J_{28}^1$
[ATP]	$-J_3^1 - J_6^1 + J_{\mathrm{gly}} - J_{\mathrm{AK}} - J_{\mathrm{hydro}} + J_{\mathrm{oxphos}} - J_{\mathrm{CK}} - J_{29}^1 - J_{30}^1$
[PCr]	J_{CK}
[AMPK]	$-J_1^1 - J_2^1 - J_3^1 - J_7^1 + J_{20}^1$
[pAMPK]	$-J_4^1 - J_5^1 - J_6^1 + J_8^1 - J_{19}^1$
[AMP–AMPK]	$J_1^1 - J_9^1 - J_{15}^1$
[ADP–AMPK]	$J_2^1 - J_{11}^1 - J_{17}^1$
[ATP–AMPK]	$J_3^1 - J_{13}^1 + J_{22}^1$
[AMP–pAMPK]	$J_4^1 + J_{10}^1 + J_{16}^1 - J_{23}^1 + J_{24}^1$
[ADP–pAMPK]	$J_5^1 + J_{12}^1 + J_{18}^1$
[ATP–pAMPK]	$J_6^1 + J_{14}^1 - J_{21}^1$
[Ca]	$-J_{\mathrm{Ca}}$
[CaM]	$-J_{\mathrm{Ca}}$
[CaCaM]	J_{Ca}
[CaMKK]	$-J_{\mathrm{CaMKK–act}} + J_{\mathrm{CaMKK–dephos}}$
[CaMKK–act]	$J_{\mathrm{CaMKK–act}} - J_{\mathrm{CaMKK–dephos}} - J_7^1 + J_8^1 - J_9^1 + J_{10}^1 - J_{11}^1 + J_{12}^1 - J_{13}^1 + J_{14}^1$
[CaMKK–act–AMPK]	$J_7^1 - J_8^1 - J_{27}^1 - J_{28}^1 - J_{29}^1$
[CaMKK–act–AMP–AMPK]	$J_9^1 - J_{10}^1 + J_{27}^1$
[CaMKK–act–ADP–AMPK]	$J_{11}^1 - J_{12}^1 + J_{28}^1$
[CaMKK–act–ATP–AMPK]	$J_{13}^1 - J_{14}^1 + J_{29}^1$
[LKB1]	$-J_{15}^1 + J_{16}^1 - J_{17}^1 + J_{18}^1$
[LKB1–AMP–AMPK]	$J_{15}^1 - J_{16}^1$
[LKB1–ADP–AMPK]	$J_{17}^1 - J_{18}^1$
[PP]	$-J_{19}^1 + J_{20}^1 - J_{21}^1 + J_{22}^1$
[PP–pAMPK]	$J_{19}^1 - J_{20}^1 - J_{30}^1$
[PP–ATP–pAMPK]	$J_{21}^1 - J_{22}^1 + J_{30}^1$
[AMPKAR]	$-J_{23}^1 + J_{26}^1$
[pAMPKAR]	$J_{24}^1 - J_{25}^1$
[AMPKAR–AMP–pAMPK]	$J_{23}^1 - J_{24}^1$
[PP1]	$-J_{25}^1 + J_{26}^1$
[PP1–pAMPKAR]	$J_{25}^1 - J_{26}^1$

Supplementary Table S22: **Equations for model 2.**

Name	ODE
[AMP]	$-J_1^2 - J_4^2 - J_{\text{AK}}$
[ADP]	$-J_2^2 - J_5^2 - J_{\text{gly}} + 2J_{\text{AK}} + J_{\text{hydro}} - J_{\text{oxphos}} + J_{\text{CK}}$
[ATP]	$-J_3^2 - J_6^2 + J_{\text{gly}} - J_{\text{AK}} - J_{\text{hydro}} + J_{\text{oxphos}} - J_{\text{CK}}$
[PCr]	J_{CK}
[Ca]	$-J_{\text{Ca}}$
[CaM]	$-J_{\text{Ca}}$
[CaCaM]	J_{Ca}
[CaMKK]	$-J_{\text{CaMKK–act}} + J_{\text{CaMKK–dephos}}$
[CaMKK–act]	$J_{\text{CaMKK–act}} - J_{\text{CaMKK–dephos}}$
[AMPK]	$-J_1^2 - J_2^2 - J_3^2 - J_7^2 + J_{13}^2$
[pAMPK]	$-J_4^2 - J_5^2 - J_6^2 + J_7^2 - J_{13}^2$
[AMP–AMPK]	$J_1^2 - J_8^2 - J_{11}^2$
[ADP–AMPK]	$J_2^2 - J_9^2 - J_{12}^2$
[ATP–AMPK]	$J_3^2 - J_{10}^2 + J_{14}^2$
[AMP–pAMPK]	$J_4^2 + J_8^2 + J_{11}^2$
[ADP–pAMPK]	$J_5^2 + J_9^2 + J_{12}^2$
[ATP–pAMPK]	$J_6^2 + J_{10}^2 - J_{14}^2$
[AMPKAR]	$-J_{15}^2 + J_{16}^2$
[pAMPKAR]	$J_{15}^2 - J_{16}^2$

Supplementary Table S23: **Equations for model 3.**

**Name**	**ODE**
[AMP]	$-J_1^3 - J_4^3 - J_{10}^3 - J_{21}^3 - J_{29}^3 - J_{40}^3 - J_{\mathrm{AK}}$
[ADP]	$-J_2^3 - J_5^3 - J_{13}^3 - J_{24}^3 - J_{32}^3 - J_{43}^3 - J_{\mathrm{gly}} + 2J_{\mathrm{AK}} + J_{\mathrm{hydro}} - J_{\mathrm{oxphos}} + J_{\mathrm{CK}}$
[ATP]	$-J_3^3 - J_6^3 - J_{16}^3 - J_{35}^3 + J_{\mathrm{gly}} - J_{\mathrm{AK}} - J_{\mathrm{hydro}} + J_{\mathrm{oxphos}} - J_{\mathrm{CK}}$
[PCr]	J_{CK}
[Ca]	$-J_{\mathrm{Ca}}$
[AMPK]	$-J_1^3 - J_2^3 - J_3^3 - J_7^3 - J_{18}^3 + J_{27}^3$
[pAMPK]	$-J_4^3 - J_5^3 - J_6^3 + J_8^3 + J_{19}^3 - J_{26}^3 - J_{37}^3 + J_{38}^3$
[AMP–AMPK]	$J_1^3 - J_9^3 - J_{20}^3 - J_{28}^3 + J_{30}^3$
[ADP–AMPK]	$J_2^3 - J_{12}^3 - J_{23}^3 + J_{33}^3$
[ATP–AMPK]	$J_3^3 - J_{15}^3 + J_{36}^3$
[AMP–pAMPK]	$J_4^3 + J_{11}^3 + J_{22}^3 - J_{39}^3 + J_{41}^3$
[ADP–pAMPK]	$J_5^3 + J_{14}^3 + J_{25}^3 - J_{31}^3 - J_{42}^3 + J_{44}^3$
[ATP–pAMPK]	$J_6^3 + J_{17}^3 - J_{34}^3$
[CaM]	$-J_{\mathrm{Ca}}$
[CaCaM]	J_{Ca}
[CaMKK]	$-J_{\mathrm{CaMKK\text{–}act}} + J_{\mathrm{CaMKK\text{–}dephos}}$
[CaMKK–act]	$J_{\mathrm{CaMKK\text{–}act}} - J_{\mathrm{CaMKK\text{–}dephos}} - J_7^3 + J_8^3 - J_9^3 + J_{11}^3 - J_{12}^3 + J_{14}^3 - J_{15}^3 + J_{17}^3$
[CaMKK–act–AMPK]	$J_7^3 - J_8^3 - J_{10}^3 - J_{13}^3 - J_{16}^3$
[CaMKK–act–AMP–AMPK]	$J_9^3 + J_{10}^3 - J_{11}^3$
[CaMKK–act–ADP–AMPK]	$J_{12}^3 + J_{13}^3 - J_{14}^3$
[CaMKK–act–ATP–AMPK]	$J_{15}^3 + J_{16}^3 - J_{17}^3$
[LKB1]	$-J_{18}^3 + J_{19}^3 - J_{20}^3 + J_{22}^3 - J_{23}^3 + J_{25}^3$
[LKB1–AMPK]	$J_{18}^3 - J_{19}^3 - J_{21}^3 - J_{24}^3$
[LKB1–AMP–AMPK]	$J_{20}^3 + J_{21}^3 - J_{22}^3$
[LKB1–ADP–AMPK]	$J_{23}^3 + J_{24}^3 - J_{25}^3$
[PP]	$-J_{26}^3 + J_{27}^3 - J_{28}^3 + J_{30}^3 - J_{31}^3 + J_{33}^3 - J_{34}^3 + J_{36}^3$
[PP–pAMPK]	$J_{26}^3 - J_{27}^3 - J_{29}^3 - J_{32}^3 - J_{35}^3$
[PP–AMP–pAMPK]	$J_{28}^3 + J_{29}^3 - J_{30}^3$
[PP–ADP–pAMPK]	$J_{31}^3 + J_{32}^3 - J_{33}^3$
[PP–ATP–pAMPK]	$J_{34}^3 + J_{35}^3 - J_{36}^3$
[AMPKAR]	$-J_{37}^3 - J_{39}^3 - J_{42}^3 + J_{46}^3$
[pAMPKAR]	$J_{38}^3 + J_{41}^3 + J_{44}^3 - J_{45}^3$
[AMPKAR–pAMPK]	$J_{37}^3 - J_{38}^3 - J_{40}^3 - J_{43}^3$
[AMPKAR–AMP–pAMPK]	$J_{39}^3 + J_{40}^3 - J_{41}^3$
[AMPKAR–ADP–pAMPK]	$J_{42}^3 + J_{43}^3 - J_{44}^3$
[PP1]	$-J_{45}^3 + J_{46}^3$
[PP1–pAMPKAR]	$J_{45}^3 - J_{46}^3$

Supplementary Table S24: **Equations for model 4.**

**Name**	**ODE**
[AMP]	$-J_{\mathrm{AK}} - J_1^4 - J_4^4$
[ADP]	$-J_{\mathrm{gly}} + 2J_{\mathrm{AK}} + J_{\mathrm{hydro}} - J_{\mathrm{oxphos}} + J_{\mathrm{CK}} - J_2^4 - J_5^4$
[ATP]	$J_{\mathrm{gly}} - J_{\mathrm{AK}} - J_{\mathrm{hydro}} + J_{\mathrm{oxphos}} - J_{\mathrm{CK}} - J_3^4 - J_6^4$
[PCr]	J_{CK}
[Ca]	$-J_{\mathrm{Ca}}$
[CaM]	$-J_{\mathrm{Ca}}$
[CaCaM]	J_{Ca}
[CaMKK]	$-J_{\mathrm{CaMKK\text{–}act}} + J_{\mathrm{CaMKK\text{–}dephos}}$
[CaMKK–act]	$J_{\mathrm{CaMKK\text{–}act}} - J_{\mathrm{CaMKK\text{–}dephos}}$
[AMPK]	$-J_1^4 - J_2^4 - J_3^4 - J_7^4 - J_{11}^4 + J_{14}^4$
[pAMPK]	$-J_4^4 - J_5^4 - J_6^4 + J_7^4 + J_{11}^4 - J_{14}^4$
[AMP–AMPK]	$J_1^4 - J_8^4 - J_{12}^4 + J_{15}^4$
[ADP–AMPK]	$J_2^4 - J_9^4 - J_{13}^4 + J_{16}^4$
[ATP–AMPK]	$J_3^4 - J_{10}^4 + J_{17}^4$
[AMP–pAMPK]	$J_4^4 + J_8^4 + J_{12}^4 - J_{15}^4$
[ADP–pAMPK]	$J_5^4 + J_9^4 + J_{13}^4 - J_{16}^4$
[ATP–pAMPK]	$J_6^4 + J_{10}^4 - J_{17}^4$
[AMPKAR]	$-J_{18}^4 - J_{19}^4 - J_{20}^4 + J_{21}^4$
[pAMPKAR]	$J_{18}^4 + J_{19}^4 + J_{20}^4 - J_{21}^4$

Supplementary Table S25: **Equations for model 5.**

Name	ODE
[AMP]	$-J_1^5 - J_4^5 - J_{\mathrm{AK}} - J_{41}^5 - J_{44}^5 - J_{47}^5 - J_{50}^5$
[ADP]	$-J_2^5 - J_5^5 - J_{\mathrm{gly}} + 2J_{\mathrm{AK}} + J_{\mathrm{hydro}} - J_{\mathrm{oxphos}} + J_{\mathrm{CK}} - J_{42}^5 - J_{45}^5 - J_{48}^5 - J_{51}^5$
[ATP]	$-J_3^5 - J_6^5 + J_{\mathrm{gly}} - J_{\mathrm{AK}} - J_{\mathrm{hydro}} + J_{\mathrm{oxphos}} - J_{\mathrm{CK}} - J_{43}^5 - J_{46}^5 - J_{49}^5 - J_{52}^5$
[PCr]	J_{CK}
[AMPK]	$-J_1^5 - J_2^5 - J_3^5 - J_7^5 + J_{24}^5 - J_{21}^5$
[pAMPK]	$-J_4^5 - J_5^5 - J_6^5 + J_8^5 - J_{23}^5 + J_{22}^5 - J_{37}^5 + J_{38}^5$
[AMP–AMPK]	$J_1^5 - J_9^5 - J_{15}^5 + J_{28}^5$
[ADP–AMPK]	$J_2^5 - J_{11}^5 - J_{17}^5 + J_{30}^5$
[ATP–AMPK]	$J_3^5 - J_{13}^5 + J_{26}^5 - J_{19}^5$
[AMP–pAMPK]	$J_4^5 + J_{10}^5 + J_{16}^5 - J_{31}^5 + J_{32}^5 - J_{27}^5$
[ADP–pAMPK]	$J_5^5 + J_{12}^5 + J_{18}^5 - J_{30}^5 - J_{33}^5 + J_{34}^5$
[ATP–pAMPK]	$J_6^5 + J_{14}^5 - J_{25}^5 + J_{20}^5 - J_{35}^5 + J_{36}^5$
[Ca]	$-J_{\mathrm{Ca}}$
[CaM]	$-J_{\mathrm{Ca}}$
[CaCaM]	J_{Ca}
[CaMKK]	$-J_{\mathrm{CaMKK–act}} + J_{\mathrm{CaMKK–dephos}}$
[CaMKK–act]	$J_{\mathrm{CaMKK–act}} - J_{\mathrm{CaMKK–dephos}} - J_7^5 + J_8^5 - J_9^5 + J_{10}^5 - J_{11}^5 + J_{12}^5 - J_{13}^5 + J_{14}^5$
[CaMKK–act–AMPK]	$J_7^5 - J_8^5 - J_{41}^5 - J_{42}^5 - J_{43}^5$
[CaMKK–act–AMP–AMPK]	$J_9^5 - J_{10}^5 + J_{41}^5$
[CaMKK–act–ADP–AMPK]	$J_{11}^5 - J_{12}^5 + J_{42}^5$
[CaMKK–act–ATP–AMPK]	$J_{13}^5 - J_{14}^5 + J_{43}^5$
[LKB1]	$-J_{15}^5 + J_{16}^5 - J_{17}^5 + J_{18}^5 - J_{19}^5 + J_{20}^5 - J_{21}^5 + J_{22}^5$
[LKB1–AMP–AMPK]	$J_{15}^5 - J_{16}^5 + J_{44}^5$
[LKB1–ADP–AMPK]	$J_{17}^5 - J_{18}^5 + J_{45}^5$
[LKB1–ATP–AMPK]	$J_{19}^5 - J_{20}^5 + J_{46}^5$
[LKB1–AMPK]	$J_{21}^5 - J_{22}^5 - J_{44}^5 - J_{45}^5 - J_{46}^5$
[PP]	$-J_{23}^5 + J_{24}^5 - J_{25}^5 + J_{26}^5 - J_{27}^5 + J_{28}^5 - J_{29}^5 + J_{30}^5$
[PP–pAMPK]	$J_{23}^5 - J_{24}^5 - J_{47}^5 - J_{49}^5 - J_{48}^5$
[PP–ATP–pAMPK]	$J_{25}^5 - J_{26}^5 + J_{49}^5$
[PP–AMP–pAMPK]	$J_{27}^5 - J_{28}^5 + J_{47}^5$
[PP–ADP–pAMPK]	$J_{29}^5 - J_{30}^5 + J_{48}^5$
[AMPKAR]	$-J_{31}^5 + J_{40}^5 - J_{33}^5 - J_{35}^5 - J_{37}^5$
[pAMPKAR]	$J_{32}^5 - J_{39}^5 + J_{34}^5 + J_{36}^5 + J_{38}^5$
[AMPKAR–pAMPK]	$J_{37}^5 - J_{38}^5 - J_{50}^5 - J_{51}^5 - J_{52}^5$
[AMPKAR–AMP–pAMPK]	$J_{31}^5 - J_{32}^5 + J_{50}^5$
[AMPKAR–ADP–pAMPK]	$J_{33}^5 - J_{34}^5 + J_{51}^5$
[AMPKAR–ATP–pAMPK]	$J_{35}^5 - J_{36}^5 + J_{52}^5$
[PP1]	$-J_{39}^5 + J_{40}^5$
[PP1–pAMPKAR]	$J_{39}^5 - J_{40}^5$

Supplementary Table S26: **Equations for model 6.**

**Name**	**ODE**
[AMP]	$-J_1^6 - J_4^6 - J_{\text{AK}}$
[ADP]	$-J_2^6 - J_5^6 - J_{\text{gly}} + 2J_{\text{AK}} + J_{\text{hydro}} - J_{\text{oxphos}} + J_{\text{CK}}$
[ATP]	$-J_3^6 - J_6^6 + J_{\text{gly}} - J_{\text{AK}} - J_{\text{hydro}} + J_{\text{oxphos}} - J_{\text{CK}}$
[PCr]	J_{CK}
[Ca]	$-J_{\text{Ca}}$
[CaM]	$-J_{\text{Ca}}$
[CaCaM]	J_{Ca}
[CaMKK]	$-J_{\text{CaMKK–act}} + J_{\text{CaMKK–dephos}}$
[CaMKK–act]	$J_{\text{CaMKK–act}} - J_{\text{CaMKK–dephos}}$
[AMPK]	$-J_1^6 - J_2^6 - J_3^6 - J_7^6 - J_{11}^6 + J_{15}^6$
[pAMPK]	$-J_4^6 - J_5^6 - J_6^6 + J_7^6 + J_{11}^6 - J_{15}^6$
[AMP–AMPK]	$J_1^6 - J_8^6 - J_{12}^6 + J_{16}^6$
[ADP–AMPK]	$J_2^6 - J_9^6 - J_{13}^6 + J_{17}^6$
[ATP–AMPK]	$J_3^6 - J_{10}^6 - J_{14}^6 + J_{18}^6$
[AMP–pAMPK]	$J_4^6 + J_8^6 + J_{12}^6 - J_{16}^6$
[ADP–pAMPK]	$J_5^6 + J_9^6 + J_{13}^6 - J_{17}^6$
[ATP–pAMPK]	$J_6^6 + J_{10}^6 + J_{14}^6 - J_{18}^6$
[AMPKAR]	$-J_{19}^6 - J_{20}^6 - J_{21}^6 - J_{22}^6 + J_{23}^6$
[pAMPKAR]	$J_{19}^6 + J_{20}^6 + J_{21}^6 + J_{22}^6 - J_{23}^6$

Supplementary References

1. Coccimiglio, I. F. & Clarke, D. C. ADP Is the Dominant Controller of AMP-activated Protein Kinase Activity Dynamics in Skeletal Muscle during Exercise. *PLOS Computational Biology* **16** (ed Beard, D. A.) e1008079. ISSN: 1553-7358 (July 2020).
2. Leung, A. & Rangamani, P. Computational Modeling of AMPK and mTOR Crosstalk in Glutamatergic Synapse Calcium Signaling. *npj Systems Biology and Applications* **9,** 1–15. ISSN: 2056-7189 (July 2023).
3. Vicini, P. & Kushmerick, M. J. Cellular Energetics Analysis by a Mathematical Model of Energy Balance: Estimation of Parameters in Human Skeletal Muscle. *American Journal of Physiology-Cell Physiology* **279,** C213–C224. ISSN: 0363-6143, 1522-1563 (July 2000).
4. Lambeth, M. J. & Kushmerick, M. J. A Computational Model for Glycogenolysis in Skeletal Muscle. *Annals of Biomedical Engineering* **30,** 808–827. ISSN: 0090-6964 (June 2002).
5. Salti, A. *et al.* High Glycolytic Activity Enhances Stem Cell Reprogramming of Fahd1-KO Mouse Embryonic Fibroblasts. *Cells* **10,** 2040. ISSN: 2073-4409 (Aug. 2021).
6. Xiao, B. *et al.* Structure of Mammalian AMPK and Its Regulation by ADP. *Nature* **472,** 230–233. ISSN: 0028-0836, 1476-4687 (Apr. 2011).
7. Segel, I. H. *Enzyme Kinetics: Behavior and Analysis of Rapid Equilibrium and Steady-State Enzyme Systems* ISBN: 978-0-471-77425-9 (Wiley, May 1975).
8. Rangamani, P., Levy, M. G., Khan, S. & Oster, G. Paradoxical Signaling Regulates Structural Plasticity in Dendritic Spines. *Proceedings of the National Academy of Sciences* **113,** E5298–E5307 (Sept. 2016).
9. Mikkola, P. *et al. Prior Knowledge Elicitation: The Past, Present, and Future* May 2023.
10. Gelman, A. *et al.* Bayesian Data Analysis Third Edition (with Errors Fixed as of 15 February 2021).
11. Zhang, L., Carpenter, B., Gelman, A. & Vehtari, A. Pathfinder: Parallel Quasi-Newton Variational Inference. *Journal of Machine Learning Research* **23,** 1–49 (2022).
12. Lizcano, J. M. *et al.* LKB1 Is a Master Kinase That Activates 13 Kinases of the AMPK Subfamily, Including MARK/PAR-1. *The EMBO Journal* **23,** 833–843. ISSN: 0261-4189, 1460-2075 (Feb. 2004).
13. Marley, A. E. *et al.* Biochemical Characterization and Deletion Analysis of Recombinant Human Protein Phosphatase 2Cα. *Biochemical Journal* **320,** 801–806. ISSN: 0264-6021, 1470-8728 (Dec. 1996).
14. Scott, J. W., Norman, D. G., Hawley, S. A., Kontogiannis, L. & Hardie, D. Protein Kinase Substrate Recognition Studied Using the Recombinant Catalytic Domain of AMP-activated Protein Kinase and a Model Substrate. *Journal of Molecular Biology* **317,** 309–323. ISSN: 00222836 (Mar. 2002).
